# From Agro-Industrial Waste to Multifunctional Nanomaterials: Antioxidant and Catalytic Properties of Iron-Based Bimetallic Nanoparticles Synthesized with *Anacardium occidentale* Pericarp Extract

**DOI:** 10.3390/antiox15101363

**Published:** 2026-10-21

**Authors:** André Felipe Câmara Amaral, Karen Chrockatt de Sá Dantas, Maria Luíza Soccio Bezerra, Vera Lúcia Perussi Polez, Luciano Paulino Silva

**Affiliations:** 1 Laboratory of Nanobiotechnology (LNANO), Embrapa Genetic Resources and Biotechnology, Parque Estação Biológica, Final W5 Norte, Brasilia 70770-917, DF, Brazil; andre.camara@embrapa.br (A.F.C.A.);; 2 Postgraduate Program in Nanoscience and Nanobiotechnology, University of Brasília (UnB), Brasilia 70910-900, DF, Brazil; 3 Biology Institute, University of Brasília (UnB), Brasilia 70910-900, DF, Brazil

**Keywords:** bimetallic oxide nanoparticles, green synthesis, *Anacardium occidentale*, oxidative stress, antioxidant activity, 4-nitrophenol reduction

## Abstract

Oxidative stress and industrial water pollution represent critical global challenges that demand the development of sustainable, multitarget remediation platforms. This study addresses the existing knowledge gap regarding the modulation of green-synthesized iron nanostructures by secondary transition metals through the fabrication of silver–iron (AgFe), cobalt–iron (CoFe), and copper–iron (CuFe) bimetallic oxide formulations using *Anacardium occidentale* L. pericarp extracts as dual-functional reducing and capping agents. Characterization was performed using UV-Vis, DLS, AFM, Raman, FTIR, and XRD techniques to elucidate their structural properties. Our results demonstrate that phytochemicals within the agro-industrial byproduct facilitated the formation of stable, spherical bimetallic oxide nanostructures. The CuFe formulation exhibited the highest catalytic activity, achieving 99.1% conversion/reduction of 4-nitrophenol within 6.3 min. Furthermore, all bimetallic oxide formulations demonstrated superior radical scavenging capacity, as evidenced by FRAP/DPPH assays, confirming the synergistic effect of the secondary metals on electron-transfer kinetics. These findings indicate that the integration of secondary metals significantly enhances the performance of biogenic iron-based oxide nanoparticles. Consequently, this study establishes an eco-friendly protocol for the production of bimetallic oxide nanostructures, providing a robust, dual-purpose platform for both environmental remediation and advanced biomedical antioxidant applications.

## 1. Introduction

The rapid expansion of global industrialization and its associated anthropogenic pressures have led to a critical intersection of environmental and biomedical crises. Among these, the contamination of aquatic ecosystems by recalcitrant organic pollutants, most notably nitroaromatic compounds such as 4-nitrophenol, presents a severe ecological threat due to their high toxicity, persistence, and bioaccumulative nature [1]. Oxidative deterioration of organic matrices, including food products, cosmetics, polymeric packaging, and industrial effluents, is driven by reactive oxygen species (ROS), such as the hydroxyl radical (OH•), the superoxide anion radical ($O_{2}^{\cdot -}$), hydrogen peroxide (H_2_O_2_), and others [2]. The high reactivity of these molecules promotes the indiscriminate oxidation of lipids, proteins and nucleic acids, resulting in loss of functionality, reduced shelf life, and the generation of toxic by-products [3,4]. Conventional synthetic antioxidants, such as butylated hydroxytoluene (BHT), propyl gallate, and tert-butylhydroquinone (TBHQ), as well as natural antioxidants (tocopherols, ascorbic acid, and carotenoids) exhibit critical physicochemical limitations for practical applications. These include poor thermal stability, accelerated photodegradation, high volatility during industrial processes, and gradual loss of activity over extended storage periods [5,6]. Such constraints motivate the search for more robust and stable alternatives. Addressing these twin challenges demands the urgent development of multifunctional, high-performance remediation platforms capable of operating under the principles of both advanced environmental catalysis and efficient antioxidant intervention.

In this context, nanotechnology provides inorganic platforms with high specific surface area, abundant active sites, and excellent durability under harsh conditions [7]. Metal and metal-oxide nanoparticles stand out for their ability to act as effective antioxidant agents, mediated by reversible surface redox pairs, which facilitate electron transfer and radical neutralization without undergoing rapid degradation [8,9].

Although monometallic nanoparticles display intrinsic antioxidant activity, bimetallic systems, particularly those based on iron combined with secondary transition metals (Ag, Co, Cu), significantly enhance this performance. The synergy between the two metals accelerates interfacial electron transfer, increases the density of catalytic sites, and amplifies the overall radical-scavenging capacity compared to their monometallic counterparts [10,11]. However, traditional chemical synthesis methods employ toxic reducing agents (sodium borohydride, hydrazine) and organic solvents, generating hazardous waste and incurring high environmental costs [12].

Plant-mediated nanofabrication utilizes the abundant phytochemicals present in various parts of plants and in agro-industrial byproducts to simultaneously reduce and stabilize metal precursors [13,14]. This biological capping layer not only prevents colloidal agglomeration but also confers additional antioxidant activity to the final nanomaterial [15].

Among the available agro-industrial coproducts, the cashew nut pericarp (*Anacardium occidentale* L.) stands out as a particularly promising feedstock. This coproduct, generated in large volumes by the cashew processing industry, is exceptionally rich in phenolic acids, anacardic acid, cardols, and cardanols, compounds with recognized reducing and chelating capacity [16]. Valorizing this coproduct aligns with circular economy principles, converting an environmental liability into a high-value technological input.

Beyond its application as a chemical antioxidant, the redox versatility of these iron-based bimetallic nanostructures extends their functionality toward environmental remediation. Heterogeneous catalysis promoted by these materials is effective in reducing persistent organic pollutants, such as 4-nitrophenol, a model contaminant widely found in industrial effluents, converting it into 4-aminophenol, a compound of lower toxicity, through electron transfer from the donor ${BH}_{4}^{-}$ [17,18]. Dual-functional nanomaterials thus optimize resource efficiency by bridging the gap between biomedical antioxidant protection and ecological pollutant degradation within a single sustainable platform [19].

The current literature lacks a systematic comparative evaluation regarding how different secondary transition metals modulate the dual biological and environmental performance of green-synthesized iron nanostructures. This investigation addresses this gap through the eco-friendly fabrication of iron-based bimetallic oxide systems. Extracts from *A. occidentale* pericarps serve as the sole reducing and capping agents for generating silver–iron (AgFe), cobalt–iron (CoFe), and copper–iron (CuFe) formulations. Comprehensive physicochemical characterizations and comparative assays were conducted to evaluate their antioxidant potential (via DPPH (2,2-Diphenyl-1-picrylhydrazyl) and FRAP (Ferric Reducing Antioxidant Power)) and their catalytic activity for 4-nitrophenol reduction. The resulting data offer a comprehensive framework for engineering multitarget nanoparticles capable of addressing biomedical and ecological challenges simultaneously.

## 2. Materials and Methods

### 2.1. Chemical Reagents and Standards

All chemical reagents, including silver nitrate PA (AgNO_3_), iron(II) sulfate heptahydrate (FeSO_4_ · 7H_2_O), iron(III) chloride (FeCl_3_), cobalt sulfate heptahydrate (CoSO_4_ · 7H_2_O), copper acetate monohydrate (Cu(CH_3_COO)_2_ · H_2_O), glacial acetic acid, sodium borohydride (NaBH_4_), 4-nitrophenol (4-NP), Folin–Ciocalteu reagent, sodium carbonate (Na_2_CO_3_), aluminum chloride (AlCl_3_), sodium acetate (CH_3_COONa), hydrochloric acid (HCl), 2,4,6-Tris(2-pyrydil)-s-triazine (TPTZ), quercetin, gallic acid, and 2,2-diphenyl-1-picrylhydrazyl (DPPH) were purchased from Sigma-Aldrich (St. Louis, MO, USA) and used without further purification. Extran^®^ 1% solution was used for glassware and biological material cleaning. Ultrapure water (Type I) and distilled water (Type III) were obtained from a GEHAKA (São Paulo, SP, Brazil) purification system.

### 2.2. Biological Material and Aqueous Extract Preparation

Cashew (*Anacardium occidentale* L.) fruits were obtained from the Active Germplasm Bank of Cashew at Embrapa Tropical Agroindustry (CNPAT, Fortaleza, CE, Brazil). The harvested samples were transported to the Nanobiotechnology Laboratory (LNANO) at Embrapa Recursos Genéticos e Biotecnologia (Cenargen, Brasília, DF, Brazil), where they were stored in cardboard boxes at room temperature until processing. For the extraction process, the fruits were washed once with a 1% Extran^®^ solution in distilled water (Type III) and cut to separate the almonds from the pericarps, which were then stored in a freezer at −20 °C until use. To prepare the aqueous extract using a green decoction approach, the frozen pericarps were thawed at room temperature and fragmented into small pieces of approximately 2 mm^2^. For the extract preparation, 1.0 g of fresh fragmented pericarps was added to approximately 50 mL of ultrapure water (Type I) (1:50, *m*/*v*) and subjected to decoction at 100 °C (monitored by a thermometer) for 2 min. The resulting mixture was filtered through an analytical funnel equipped with blue-band qualitative filter paper (average pore size of 14 µm), and the resulting aqueous extract was adjusted to a final concentration of 20 mg mL^−1^ (based on fresh pericarp mass per volume of water), which was determined gravimetrically. The fresh aqueous extracts were utilized immediately for phytochemical assays and nanoparticle synthesis.

### 2.3. Phytochemical Characterization of the Extract

#### 2.3.1. Total Phenolic Content (TPC)

The TPC of the aqueous extract was determined using an adapted version of the Folin–Ciocalteu colorimetric method, as previously described by Silveira et al. [20]. Briefly, a 20 µL aliquot of the fresh extract was diluted with 130 µL of distilled water. Subsequently, 50 µL of the Folin–Ciocalteu reagent and 100 µL of a 20% sodium carbonate (Na_2_CO_3_) solution were added to promote the reaction with phenolic compounds. After an incubation period of 30 min at room temperature, the samples were transferred to a 96-well microplate and absorbance was recorded at 630 nm using a microplate reader (Uniscience, Osasco, SP, Brazil). A calibration curve (y = 0.18x − 5.87 × 10^−3^, R^2^ = 1) was established using gallic acid (1 × 10^−4^ mol L^−1^) as the reference standard. The final results were expressed as milligrams of gallic acid equivalents per gram of sample (mg GAE g^−1^).

#### 2.3.2. Total Flavonoid Content (TFC)

The TFC was quantified using the aluminum chloride (AlCl_3_) colorimetric assay, adapted from Sembiring et al. [21] with minor modifications. Quercetin standard solutions were prepared in 96% ethanol across a concentration range of 30 to 100 µg mL^−1^ (30, 40, 50, 60, 70, 80, 90, and 100 µg mL^−1^). For the reaction, 50 µL of the pericarp aqueous extract (20 mg mL^−1^) or the quercetin standard solutions was mixed with 10 µL of a 10% (*w*/*v*) AlCl_3_ solution in a 96-well microplate, followed by the addition of 150 µL of 96% ethanol. Finally, 10 µL of 1 M sodium acetate was introduced into the mixture. A reagent blank consisting of 96% ethanol instead of the sample or standard was prepared under identical conditions. The reaction mixtures were thoroughly mixed and incubated at room temperature for 40 min in the dark. Absorbance values were measured at 405 nm using a microplate reader (Uniscience, Osasco, SP, Brazil). TFC values were determined from the quercetin calibration curve and expressed as milligrams of quercetin equivalents per gram of sample (mg QE g^−1^).

### 2.4. In Vitro Antioxidant Activity Assays

#### 2.4.1. Ferric Reducing Antioxidant Power (FRAP) Assay

The total antioxidant capacity based on ferric iron reduction was determined using an adapted FRAP assay [22]. Starting from the prepared aqueous extract, at least three distinct sequential dilutions were prepared in triplicate within microtubes. In a dark environment, a 35 µL aliquot of each extract dilution was transferred to test microtubes, mixed with 265 µL of freshly prepared FRAP reagent. The solutions were thoroughly homogenized using a vortex mixer and incubated in a water bath at 37 °C. After exactly 30 min of incubation, the absorbance was measured at 630 nm using a microplate reader (Uniscience, Osasco, SP, Brazil), calibrated against the FRAP reagent (y = 0.00020655x + 0.0896, R^2^ = 0.9967) without any sample as a blank and using ascorbic acid for comparison. To calculate the total antioxidant activity, a linear regression curve was plotted with absorbance on the Y-axis and sample concentration (µM) on the X-axis to determine the straight-line equation. The absorbance value equivalent to 1 mM of the ferrous sulfate (FeSO_4_ · 7H_2_O) standard was substituted into the equation to determine the term x, which represents the sample dilution (mg L^−1^) corresponding to the standard reduction power. The final antioxidant activity (Z) was calculated by dividing 1 mM by the mass conversion of X (in grams) and normalized to g, expressing the results as micromoles of ferrous sulfate equivalent per gram of sample (µM FeSO_4_ g^−1^).

#### 2.4.2. DPPH Radical Scavenging Assay

The free radical scavenging capability of the extract was evaluated using the 2,2-diphenyl-1-picrylhydrazyl (DPPH) radical assay, following the protocol adapted from Polez [23]. A 30 µL aliquot of the aqueous extract was diluted in 120 µL of distilled water and mixed with a methanolic solution of DPPH (0.06 mM). The mixture was vortexed thoroughly and allowed to incubate in the dark for 30 min. Afterward, the samples were vortexed again and centrifuged at 4500× *g* for 2 min. The entire sample was then returned to dark incubation to complete a total reaction time of 60 min. The final absorbance was recorded at 492 nm using a microplate reader (Uniscience, Osasco, SP, Brazil), and the results were expressed as the percentage of DPPH radical inhibition (% inhibition).

### 2.5. Fourier-Transform Infrared Spectroscopy (FTIR-ATR)

The functional groups and surface interactions of the lyophilized aqueous cashew extract were analyzed using Fourier-transform infrared spectroscopy (FTIR). The spectra were recorded using a Varian 640-IR FTIR spectrometer (Varian, Inc., Randolph, MA, USA) operating in Attenuated Total Reflectance (ATR) mode equipped with a germanium crystal. A sufficient mass of the lyophilized extract was placed directly onto the ATR system to completely cover the crystal surface. Spectra were acquired in transmittance mode within the wavenumber range of 4000 to 600 cm^−1^, employing an average of 32 scans at a spectral resolution of 4 cm^−1^. Prior to each measurement, the ATR crystal was thoroughly cleaned with analytical grade isopropyl alcohol, and ambient atmospheric air was collected as the background reference.

### 2.6. Green Synthesis of Bimetallic Oxide Nanoparticles (BNPs)

The biological fabrication of bimetallic nanoparticles (BNPs) was carried out using a sequential reduction mechanism using the *A. occidentale* L. pericarp aqueous extract as both the reducing and stabilizing agents. The metal precursor solutions consisted of aqueous formulations of silver nitrate (AgNO_3_), iron(II) sulfate (FeSO_4_), cobalt sulfate (CoSO_4_), and copper acetate (Cu(CH_3_COO)_2_). The synthesis yields mixed-metal oxide phases, as confirmed by XRD and Raman spectroscopy. In a typical experimental procedure to obtain the iron-oxide core nanomaterials, 2.5 mL of a 4 mmol L^−1^ iron(II) sulfate solution was added to 20 mL test tubes containing 2.5 mL of the cashew pericarp aqueous extract (20 mg mL^−1^). These initial reaction mixtures were incubated in a water bath at 75 °C for 240 min to ensure core formation. Following this first stage, an additional 2.5 mL aliquot of the fresh cashew aqueous extract was added to the mixture, immediately followed by the introduction of 2.5 mL of the secondary metal salt solutions (AgNO_3_, CoSO_4_, or Cu(CH_3_COO)_2_) at 4 mmol L^−1^. The secondary reduction step was maintained under water bath incubation at 75 °C for an additional 240 min. Reaction controls were simultaneously prepared: an extract control containing ultrapure water (Type I) combined with the biological extract, and salt controls (CM) comprising ultrapure water mixed with the respective metal salt solutions at a final concentration of 1 mmol L^−1^ each. After synthesis, the nanoparticle suspensions were purified by three sequential centrifugation–washing cycles for the antioxidant assay. Each cycle consisted of centrifugation at 16,100 RCF for 20 min at 22 °C, removal of the supernatant, and redispersion of the pellet in ultrapure water (Type I) in the same volume as the original suspension. The supernatant of the first cycle (SN) was kept for analysis, and the pellet obtained after the last cycle was used as the washed nanoparticles. The FRAP and DPPH assays (Section 2.4.1 and Section 2.4.2) were applied to the extract, the as-synthesized suspensions, the SN and the washed nanoparticles, using the same aliquot volume for each sample.

### 2.7. Nanoparticle Characterization Techniques

#### 2.7.1. UV-Vis Spectrophotometry

The optical properties and surface plasmon resonance transitions of the synthesized bimetallic oxide nanoparticles were monitored using a Q898U (Quimis, Diadema, SP, Brazil) ultraviolet–visible (UV-Vis) spectrophotometer. After a 24 h stabilization period post-synthesis, a complete spectral sweep was recorded in the range of 300 to 550 nm using sample aliquots diluted in a 1:10 ratio (BNPs: H_2_O, *v*/*v*).

#### 2.7.2. Dynamic Light Scattering (DLS) and Electrophoretic Mobility

The hydrodynamic diameter (d_H_, Z-average), polydispersity index (PdI), and Zeta potential (ζ) of all synthesized bimetallic oxide nanoparticles were determined after reaction using a Malvern ZetaSizer Nano system (Malvern, UK). For the analyses, 100 µL of the BNP solution was mixed with 900 µL of ultrapure water (Type I) inside polypropylene microtubes, homogenized, and transferred into DTS 1070 folded capillary cells. The measurement parameters were standardized as follows: operations performed in triplicate, an equilibration time of 120 s prior to data acquisition, a constant cell temperature of 25 °C, a He-Ne laser source (λ = 632.8 nm), and a fixed scattering angle of 173°. Automatic run adjustments (number and duration of acquisitions) were managed by the ZetaSizer software (version 7.11).

#### 2.7.3. Atomic Force Microscopy (AFM)

The topography, shape, and height distribution of the synthesized nanomaterials were investigated using a Shimadzu SPM-9600 atomic force microscope (Shimadzu, Kyoto, Japan). The system was operated in dynamic-viscoelastic mode utilizing a conical silicon tip with a tip radius below 10 nm and a rectangular cantilever featuring a spring constant of approximately 42 N m^−1^ and a resonant frequency of around 260 kHz. Sample preparation involved diluting the bimetallic oxide nanoparticle suspensions in ultrapure water at a 1:20 ratio, after which a 1 µL aliquot was deposited onto a freshly cleaved muscovite mica substrate mounted on a metallic sample holder with double-sided adhesive tape. Scan areas of 10 µm × 10 µm were selected randomly, collecting both height and phase images at a scan rate of 1 Hz and a spatial resolution of 512 × 512 lines. Image flattening and X-axis leveling were performed offline using Gwyddion version 2.69 software. Size distribution modeling was conducted through Gaussian function fittings utilizing Microsoft Excel (Office 365).

#### 2.7.4. Raman Spectroscopy

The structural arrangement and crystalline phase boundaries of the bimetallic nanomaterials were probed using Confocal Raman Spectroscopy. The distinct bimetallic oxide nanoparticle suspensions were prepared by dropping 5 successive aliquots of 1 µL onto specific spots of a stainless-steel microplate, allowing complete air-drying between each deposition. The evaluations were executed using an Alpha 300 RA confocal Raman microscope (WITec, Ulm, Germany) coupled to a TOPTICA SYS XTRA diode laser module operating at an excitation wavelength of 785 nm and a maximum output power of 300 mW. Single-spectrum acquisitions were conducted using a 100× objective lens, a 785 nm holographic edge filter, and a charge-coupled device (CCD) camera cooled to −60 °C. The acquisition setup employed an integration time of 0.2 s per frame with 100 accumulations. To ensure statistical representativeness, at least three separate regions were scanned per sample, and their spectral signals were averaged to generate the final representative spectrum.

#### 2.7.5. X-Ray Diffraction Analysis

The crystalline structure of the synthesized nanoparticles was investigated by X-ray diffraction (XRD) using a Bruker D8 Focus diffractometer equipped with Cu Kα radiation (λ = 1.5406 Å). The diffraction patterns were recorded over a 2θ range of 10–90°, using a step size of 0.02° and a counting time of 2 s per step.

Approximately 50 mg of each nanoparticle sample were dried in an oven and subsequently ground using an agate mortar to obtain a homogeneous powder. The powdered samples were then evenly deposited onto a glass sample holder for XRD analysis. The mean crystallite size (*D*) was estimated from the most intense diffraction peak of each sample using the Scherrer equation:

$$D=\frac{K\lambda }{\beta \cos\theta }$$

where *D* is the mean crystallite size, *K* is the shape factor (0.9), *λ* is the Cu Kα wavelength (1.5406 Å), *β* is the full width at half maximum (FWHM) of the selected diffraction peak expressed in radians, and *θ* is the Bragg angle corresponding to the selected reflection.

### 2.8. Evaluation of Catalytic Performance (4-Nitrophenol Reduction)

The catalytic remediation efficiency of the green-synthesized bimetallic oxide nanoparticles was evaluated through the model chemical reduction of 4-nitrophenol (4-NP) to 4-aminophenol (4-AP) in the presence of sodium borohydride (NaBH_4_). The liquid-phase reactions were conducted statically at room temperature within flat-bottomed 96-well polystyrene microplates and monitored using inline UV-Vis spectrophotometry. In a standard protocol, 1 µL of the respective bimetallic oxide catalyst suspension was pipetted into a well, followed by the addition of a 100 µL reaction cocktail composed of 50 µL of 4-NP (0.5 mmol L^−1^) and 50 µL of a freshly prepared NaBH_4_ solution (50 mmol L^−1^). To prevent spontaneous thermal decomposition and maintain reducing stability, the NaBH_4_ stock solution was kept in an ice bath prior to use. The reaction progress was tracked by measuring the decay of the target absorption band at 405 nm, which corresponds to the characteristic electronic transition of the 4-nitrophenolate intermediate ion formed under alkaline conditions. Kinetic profiles were established by converting raw absorbance data into concentration over time based on a standard calibration curve. The calibration curve was constructed in triplicate by measuring the absorbance of 11 distinct volumetric dilutions (ranging from 0 to 100 µL) sampled from a 0.5 mM 4-NP stock solution. Given that the initial concentration of NaBH_4_ was kept in vast excess (100-fold) relative to 4-NP, its consumption rate was considered negligible, allowing the reaction to be treated as a pseudo-first-order kinetic process. The apparent reaction rate constant (k_obs_, s^−1^), total conversion time, and chemical conversion efficiency were determined from the first-order integrated rate law plots.

## 3. Results and Discussion

### 3.1. Characterization of Anacardium occidentale L. Fruit Extract (Pericarp)

The quantitative and qualitative evaluation of the biogenic matrix is a fundamental step in green nanotechnology, as the secondary metabolites govern both the reduction kinetics of metal precursors and the subsequent stabilization of the nanomaterials. The aqueous extract obtained from *A. occidentale* pericarps demonstrated a total phenolic content (TPC) of 377.59 ± 27.69 mg GAE g^−1^ and a total flavonoid content (TFC) of 69.30 ± 5.08 mg QE g^−1^. These values were higher than those reported for other cashew by-products. For instance, cashew apple bagasse extracts obtained using microwave-assisted extraction (41.01 ± 2.11 mg GAE g^−1^) [24] or ultrasound-assisted extraction (~30 mg GAE g^−1^) [25] showed TPC values approximately 9- to 12-fold lower than that obtained in the present study. Likewise, a recent study evaluating tropical fruit peels reported the highest TPC and TFC values for passion fruit peel (MPP), at 69.81 ± 1.93 mg GAE g^−1^ and 29.61 ± 0.92 mg QE g^−1^, respectively [26]. Under the conditions investigated, the extract evaluated in the present study exhibited a TPC more than fivefold higher and a TFC more than twofold higher than those reported for passion fruit peel. Plant-derived polyphenols and flavonoids are well-documented for their robust antioxidant and electron-donating properties. In the context of green synthesis, these biomolecules act as excellent sacrificial reducing agents, capable of donating electrons to convert multivalent metal ions (Fe^2+^, Ag^+^, Co^2+^, and Cu^2+^) into zero-valent species or stable tailored metal oxide matrices. Concurrently, the oxidized forms of these phytochemicals adsorb onto the nucleated crystal surfaces, serving as capping agents that restrict uncontrolled particle growth.

**Figure 1:**
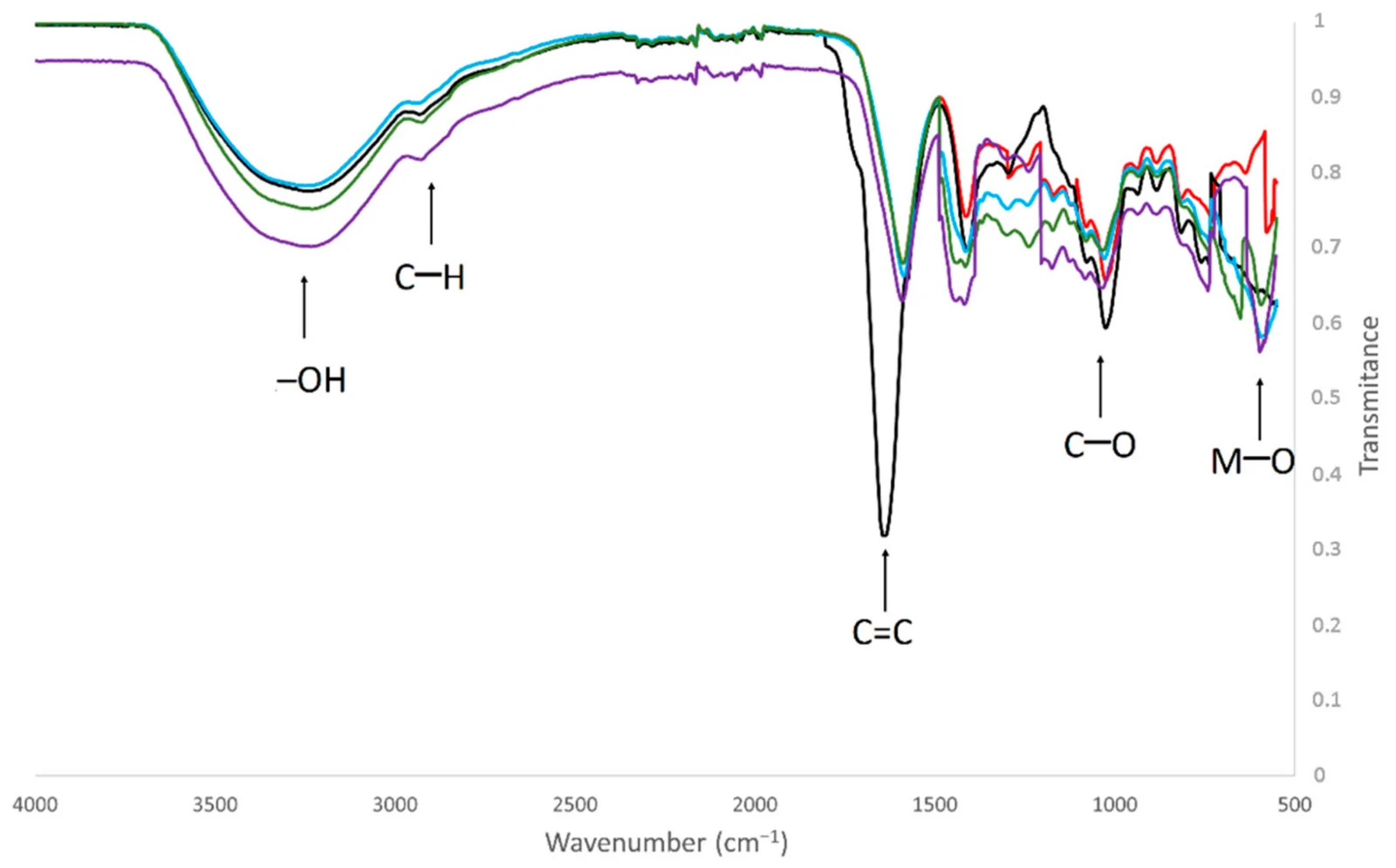
Comparative FTIR spectra of pure *A. occidentale* extract (black line) and synthesized Fe_2_O_3_ (purple line), AgFe (red line), CoFe (blue line), and CuFe (green line). The spectra are presented in transmittance mode (0–1). Arrows indicate the main absorption bands: –OH (~3361 cm^−1^), C–H (~2924 and ~2854 cm^−1^), C=C (~1604 cm^−1^), C–O (~1016 cm^−1^), and M–O (~400–700 cm^−1^).

To elucidate the specific functional groups actively involved in this bioreduction and capping phenomenon, Fourier-transform infrared spectroscopy (FTIR-ATR) was conducted on the lyophilized pericarp extract (Figure 1). The resulting spectrum revealed a complex, multifunctional chemical architecture characterized by several prominent absorption bands.

A broad and intense absorption band centered at 3361 cm^−1^ is attributed to the axial stretching vibrations of hydroxyl (–OH) groups. This band is consistent with the abundance of phenolic compounds and flavonoids, which are heavily engaged in intermolecular and intramolecular hydrogen bonding networks. The weak but well-defined absorption peaks observed at 2924 cm^−1^ and 2854 cm^−1^ correspond to the asymmetric and symmetric C–H stretching deformations of aliphatic CH_2_/CH_3_ groups, respectively, reflecting the presence of aliphatic hydrocarbon chains from native lipids, waxes, or organic acids within the pericarp.

Furthermore, a sharp and structurally significant peak emerged at 1604 cm^−1^, which is assigned to the skeletal stretching vibrations of aromatic rings (Ar–H). This feature is highly characteristic of complex aromatic networks found within condensed tannins, flavonoids, and saponins, which are ubiquitous in the *A. occidentale* matrix. The band at 1403 cm^−1^ is associated with the bending and torsion modes of –CH_2_ groups, typically originating from phenolic frameworks, as well as minor lipid or protein fractions. Additionally, the prominent and sharp absorption peak at 1016 cm^−1^ is ascribed to C–O stretching vibrations, which commonly represent glycosidic linkages in flavonoids, amino acid residues, and polymeric carbohydrates. Finally, the low-frequency bands recorded at 775 cm^−1^ and 709 cm^−1^ are indicative of out-of-plane aromatic ring bending vibrations, confirming the persistence of stable benzenoid structures.

These spectroscopic assignments align closely with recent literature data for cashew-based matrices. Specifically, Pradeep et al. [27] identified a highly comparable vibrational profile when analyzing dehydrated cashew fruits, noting a broad hydroxyl band at 3401 cm^−1^ and a distinct aliphatic stretching peak at 2925 cm^−1^ representing the C–H stretching of alkenes, alongside intense bands at 1665 cm^−1^ (C = C alkene stretching) and 1020 cm^−1^ (C–O–O–C–O anhydride stretching). The minor low-frequency shifts observed in our fresh pericarp extract (e.g., from 3401 to 3361 cm^−1^ for the—OH group) suggest a stronger network of hydrogen bonds and a denser concentration of polyhydroxy-rich components achieved using the aqueous decoction approach.

From a mechanistic perspective, the high density of these surface-active functional groups, particularly the phenolic hydroxyls and aromatic rings, is a vital prerequisite for the physical stability of the resulting bimetallic systems. As discussed by Mata et al. [28], the existence of active—OH groups within biogenic extracts is considered absolutely essential for green-synthesized nanoparticles to exhibit long-term colloidal stability. During the reaction, these hydroxyl groups not only drive the chemical reduction in the metallic precursors via electron transfer but also coordinate directly with the surface atoms of the newly formed bimetallic cores. This interfacial binding wraps the nanoparticles in a dense organic shell, generating strong steric hindrance and electrostatic repulsion that counteract attractive van der Waals forces. Consequently, the phytochemical composition of the *A. occidentale* L. pericarp extract provides an optimal, highly sustainable platform for synthesizing stable, non-agglomerated bimetallic nanomaterials without requiring hazardous synthetic surfactants.

In addition to the organic functional groups discussed above, the FTIR spectra of all bimetallic oxide formulations exhibit characteristic absorption bands in the 400–700 cm^−1^ region, which are assigned to metal-oxygen (M-O) stretching vibrations. The presence of these bands are consistent with the formation of metal oxide phases, consistent with the XRD and Raman results discussed below [29].

### 3.2. Synthesis and Characterization of Bimetallic Oxide Nanoparticles

The evaluation of optical properties using UV-Vis spectrophotometry is a widely accepted technique to confirm the formation, growth, and surface characteristics of bimetallic oxide nanomaterials synthesized through biogenic routes. The reduction in metal precursors mediated by the *A. occidentale* pericarp extract was primarily monitored by noticeable visual color alterations in the reaction mixtures, moving from the light yellowish-brown tone of the pure plant extract to dense dark brown and black suspensions after the sequential thermal incubation. This macroscopic transition provides immediate qualitative evidence of the excitation of surface plasmon resonance (SPR) transitions, signaling the collaborative reduction in the localized metal ions and the subsequent formation of mixed-metal oxide phases.

**Figure 2:**
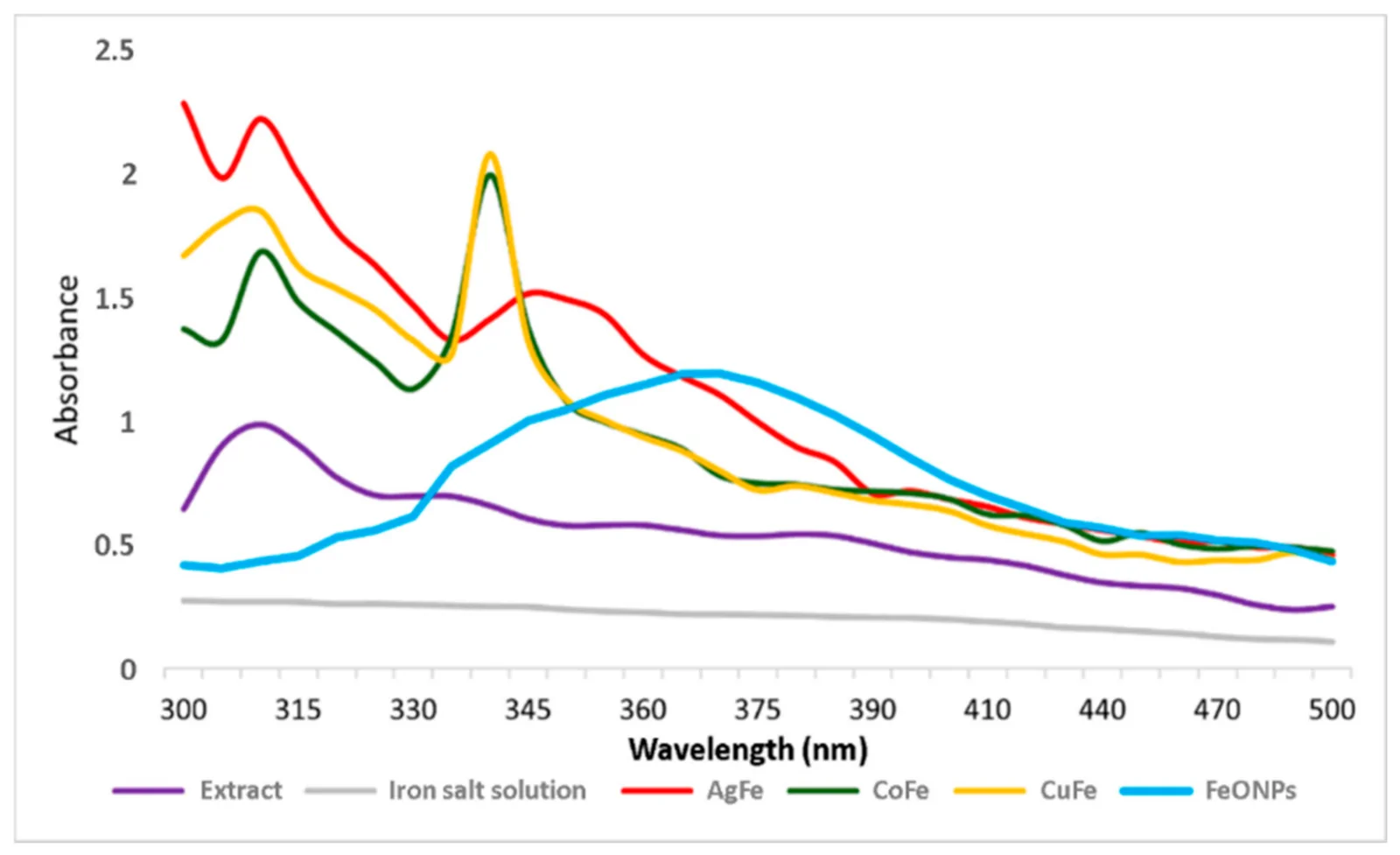
Comparative UV-Vis spectra of pure *A. occidentale* extract (purple line), salt solution (gray line), synthesized FeONPs (blue line), AgFe (red line), CoFe (green line), and CuFe (yellow line). The spectra were recorded in the 300–550 nm range.

To confirm that these changes stemmed from the assembly of bimetallic nanostructures rather than simple metal salt complexation or extract degradation, the UV-Vis absorption spectra of the individual precursor salt solutions (controls), the raw aqueous extract, and the final synthesized bimetallic nanoparticles (BNPs) were recorded and contrasted (Figure 2). The pure *A. occidentale* pericarp extract displayed its characteristic electronic transitions in the ultraviolet region, with notable shoulders and peaks below 350 nm. In contrast, the individual control metal salt formulations exhibited negligible or distinct baseline absorption bands within the visible range (350 to 550 nm), confirming their stable ionic state in the absence of a biological reducing medium.

Upon reaction completion, the UV-Vis spectra of the AgFe, CoFe, and CuFe colloidal systems underwent a significant modification. All three bimetallic oxide formulations demonstrated a broad, continuous increase in absorbance across the entire visible wavelength range (300 to 500 nm) compared to the baseline solutions. The FeONPs exhibited a characteristic absorption profile with a maximum at approximately 360 nm. For the AgFe bimetallic oxide system, a broad absorption feature was superimposed over the typical decaying scattering profile, indicating dampening and overlapping of the individual silver SPR peak with the continuous absorption of the underlying iron oxide matrix. The damping and broadening of the Ag SPR band may reflect overlap with the strong absorption of the iron oxide matrix and/or electronic interaction between Ag^0^ and the iron oxide; UV-Vis alone cannot distinguish these possibilities, and definitive proof of intimate Ag/α-Fe_2_O_3_ contact would require XPS or HR-TEM.

Similarly, the CoFe and CuFe nanoparticles displayed enhanced optical densities and broad absorption, with narrow bands at ~310 and ~340 nm that are absent in FeONPs. These bands may be related to Co/Cu oxide phases or Fe–polyphenol species, but their assignment requires further study; phase identification was based on XRD and Raman.

The absence of unreacted precursor salt peaks, combined with the prominent baseline rise and the continuous absorption profile, is consistent with the extract promoting the conversion of the metal ions (via complexation, hydrolysis/precipitation and, for Ag^+^ and Cu^2+^, reduction) and chemical precipitation of the metallic ions into stable bimetallic oxide nanostructures.

To gain deeper insights into the physical dimensions, structural homogeneity, and physical stability of the biogenically synthesized nanomaterials in aqueous suspension, dynamic light scattering (DLS) and electrophoretic mobility measurements were systematically performed. The experimental values obtained for the hydrodynamic diameter (d_H_, Z-average), polydispersity index (PdI), and Zeta potential (Zp) of the AgFe, CoFe, and CuFe bimetallic oxide nanoparticles are summarized in Table 1.

The DLS analysis revealed multimodal size distributions for all formulations, with three distinct populations observed for each system (Figure S1, Supplementary Materials). This behavior is consistent with the heterogeneous nucleation pathways typical of green synthesis using complex plant extracts, where multiple phytochemicals act as reducing and capping agents, leading to competitive nucleation and growth [30,31]. The bimetallic material and the nature of the secondary metal precursor exerted a clear influence on the spatial properties of the resulting nanostructures. The AgFe exhibited the smallest average hydrodynamic size (193.7 ± 23.2 nm), alongside a moderate polydispersity index (0.375 ± 0.020). In contrast, the sequential deposition of cobalt led to the formation of significantly larger clusters in the CoFe system, which reached a Z-average of 317.7 ± 13.0 nm and a higher PdI value (0.573 ± 0.010). The CuFe system occupied an intermediate position, displaying a hydrodynamic diameter of 205.2 ± 20.2 nm and a PdI of 0.470 ± 0.050. It is crucial to highlight that the d_H_ values measured by DLS reflect the global size of the metallic core combined with the surrounding hydration layer and the organic capping shell composed of adsorbed plant macromolecular conjugates.

The relatively broad particle size distributions (PdI > 0.3) observed across all systems, particularly in the cobalt and copper formulations, are heavily coupled with the multi-step nature of the green synthesis protocol and provide evidence of particle agglomeration in the aqueous medium. Similar PdI values have been reported for other green-synthesized bimetallic oxide systems [30,32]. For instance, high PDI values have also been reported by Hendi et al. for the bimetallic synthesis of Ag/Au [30] and by Aldahasi et al. for the synthesis of ZnO-Mn nanoparticles [32]. Unlike monometallic green syntheses, the sequential introduction of distinct transition metal ions into a highly complex biogenic matrix containing multiple active metabolites can trigger competitive reduction and nucleation paths. This frequently leads to heterogeneous layer deposition and partial cluster aggregation, a phenomenon widely documented for multimetallic green platforms.

Regarding surface characteristics, all three bimetallic systems developed a net negative surface charge, with Zeta potential values recorded at −17.5 ± 0.8 mV (AgFe), −16.3 ± 1.5 mV (CoFe), and −8.6 ± 2.6 mV (CuFe). The zeta potential values indicate moderate electrostatic repulsion, which is consistent with values reported for other green-synthesized bimetallic systems [33,34]. However, colloidal stability is maintained through a combination of electrostatic repulsion and steric hindrance provided by the high-molecular-weight organic corona confirmed by FTIR analysis (Figure 1). As established during the FTIR evaluations, the *A. occidentale* pericarp extract is rich in polyphenols, flavonoids, and condensed tannins. Under the synthesis conditions, the deprotonation of hydroxyl (–OH) and carboxylic groups belonging to these capping agents imparts a structural negative charge to the outer layer of the nanoparticles.

The low absolute Zeta potential values obtained, particularly for the CuFe system (−8.6 mV), confirm that electrostatic repulsion is insufficient to overcome the attractive van der Waals forces and magnetic dipole–dipole interactions inherent to iron-based oxide matrices. Consequently, instead of promoting ideal isolated capping, the high-molecular-weight biomolecules from the *A. occidentale* pericarp extract form an entangled polymeric network that physically entraps clusters of primary nanoparticles into stable, hydrated supramolecular agglomerates. This behavior is consistent with the mixed-metal oxide nature of the materials. This collective behavior perfectly explains the high hydrodynamic volume and the multimodal distribution profiles in the liquid phase, bridging the gap between solution dynamics and dry-state morphology.

**Figure 3:**
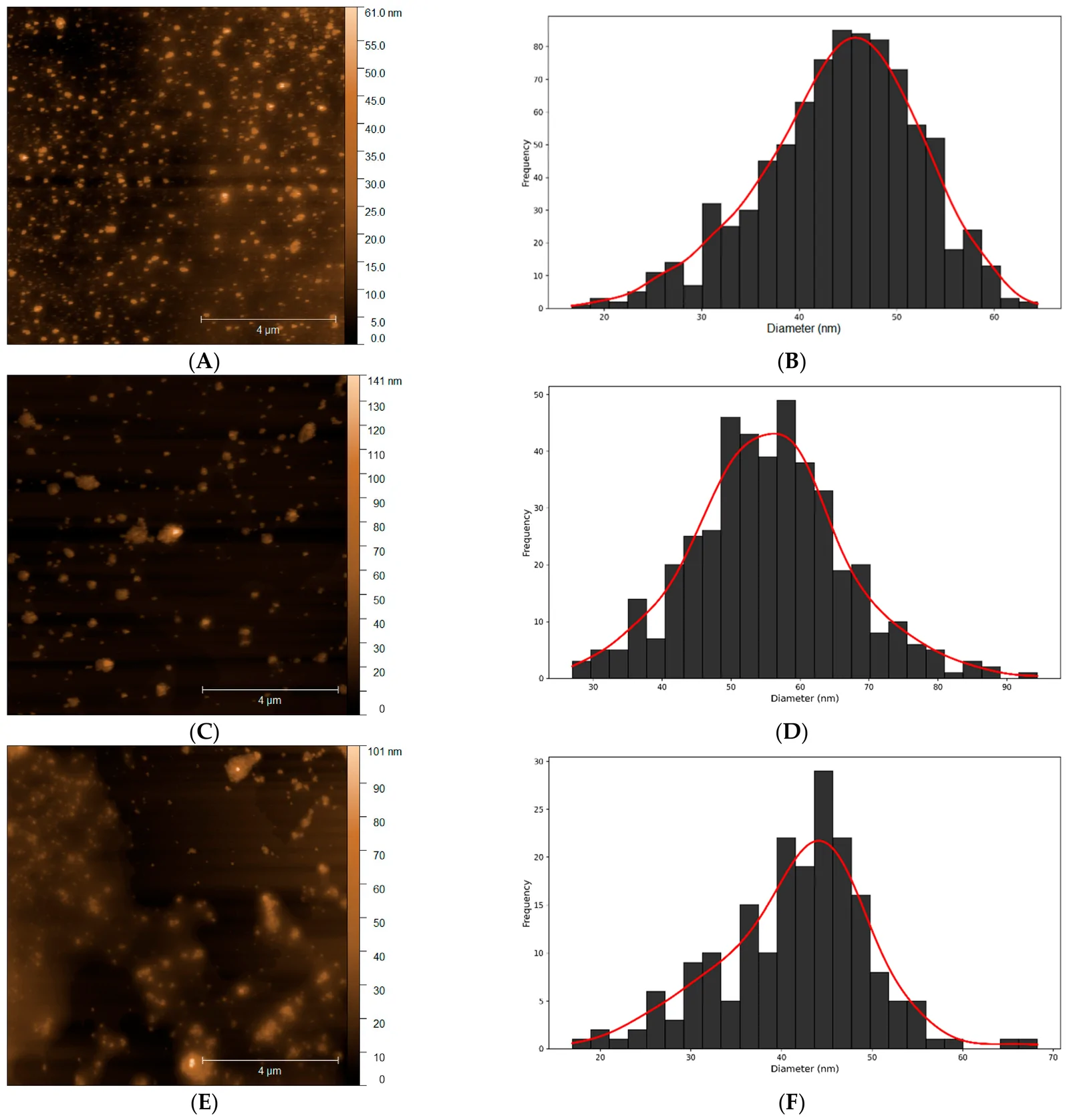
AFM panels showing 2D height map topography and Gaussian-fitted (red line) size histograms for (**A**,**B**) AgFe, (**C**,**D**) CoFe, and (**E**,**F**) CuFe bimetallic oxide nanoparticles, respectively. Scan areas: 10 µm × 10 µm.

Atomic force microscopy (AFM) was employed as a high-resolution technique to examine the three-dimensional surface topography, vertical height profiles, and physical sizes of the dry bimetallic oxide nanoparticles after deposition onto cleaved muscovite mica substrates. The 3D topographical maps, along with their respective Gaussian size distribution histograms for the AgFe, CoFe, and CuFe nanoparticle formulations, are shown in Figure 3.

The AFM micrographs revealed distinct nanoscale features, surface roughness, and aggregation states that varied according to the chemical composition of the secondary transition metal. For the AgFe bimetallic oxide system (Figure 3A,B), the nanostructures exhibited a well-dispersed, quasi-spherical, and nodular morphology. Statistical modeling of the particle heights using a Gaussian distribution revealed a mean physical core diameter of 44.1 nm. This solid-state dimension stands in sharp contrast to the average hydrodynamic diameter of 193.7 ± 23.2 nm recorded previously using DLS in aqueous suspension.

Similarly, the CuFe bimetallic oxide system (Figure 3E,F) displayed a highly homogeneous distribution of compact, uniform granular domains. The corresponding Gaussian fitting calculated the smallest mean physical size among all samples, centered at 41.5 nm. Much like the silver–iron system, this physical core boundary is substantially smaller than its fluid-state hydrodynamic counterpart (205.2 ± 20.2 nm). This recurring discrepancy between techniques highlights a well-known physical principle in green nanotechnology. While DLS measures the global, dynamic entity in a liquid environment, which includes the metallic cluster, its surrounding solvent shell, and the bulky, hydrated organic plant macroconjugates (polyphenols and flavonoids) wrapped around it, AFM scans the dry, condensed physical core boundaries after the removal of volatile solvent layers.

Conversely, the CoFe bimetallic oxide formulation (Figure 3C,D) showed a significantly different topographical behavior, characterized by the assembly of interconnected vertical aggregates and larger fused cluster domains. The corresponding analysis yielded a larger mean physical size of 55.4 nm. This larger physical boundary supports the substantial hydrodynamic size (317.7 ± 13.0 nm) and high polydispersity index (0.573 ± 0.010) observed during the light scattering assays. The multicentric growth of the cobalt oxide phases over the initial iron seeds, combined with magnetic dipole–dipole attractions common to iron and cobalt systems, likely overcomes the steric repulsion barriers during the solvent evaporation phase. This causes the nanoparticles to settle into microaggregate clusters on the substrate. Ultimately, the high-resolution AFM data confirm the green synthesis mediated by *A. occidentale* pericarp extract produces stable transition-metal oxide nanostructures with physical cores below 60 nm, whose morphology and aggregation behavior can be tailored through specific bimetallic combinations.

The observed discrepancy between DLS (Z-average: 193.7–317.7 nm) and AFM (physical core: 41.5–55.4 nm) is fully consistent with the behavior of green-synthesized bimetallic oxide nanoparticles, where the hydrodynamic diameter reflects not only the metallic core but also the tightly bound organic corona and its associated hydration layer [35,36,37]. The AFM dry-state measurements, by contrast, provide the dimension of the inorganic core after removal of the solvent and the collapse of the organic shell. This large difference confirms the presence of a thick, hydrated phytochemical coating on the nanoparticle surface, which is essential for colloidal stability.

**Figure 4:**
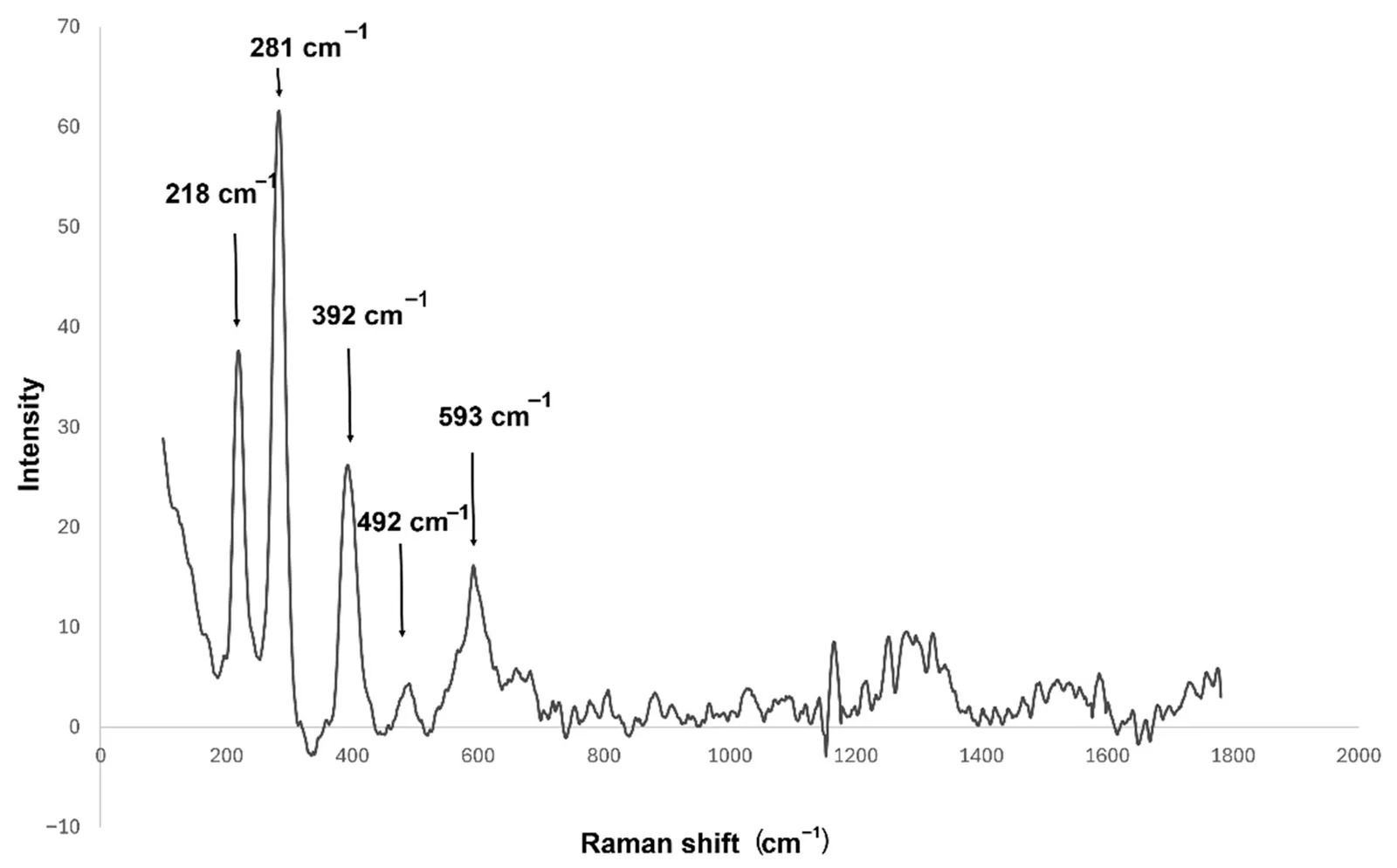
Raman scattering spectrum of AgFe bimetallic oxide nanoparticles showing characteristic bands of α-Fe_2_O_3_ (hematite) at 218, 281, 392, 492, and 593 cm^−1^.

Raman spectroscopy was employed as a powerful non-destructive analytical tool to resolve the structural arrangement, local symmetry, and crystalline phase boundaries of the green-synthesized nanomaterials. By probing local vibrational modes, this technique provided a detailed assessment of the short-range order within the crystal lattices. The Raman scattering spectrum of the AgFe bimetallic oxide nanoparticles recorded across the 100–1700 cm^−1^ wavenumber range is displayed in Figure 4.

The structural characterization of the AgFe revealed a distinct vibrational profile with well-defined scattering bands centered at 218, 281, 392, 492, and 593 cm^−1^. This specific fingerprint confirms the dominant presence of iron oxide crystalline domains in the hematite phase (α-Fe_2_O_3_). Notably, the absence of high-intensity scattering bands in the 668 cm^−1^ region suggests that the magnetite phase (Fe_3_O_4_) did not form or stabilize in concentrations detectable by this technique [38].

The recorded bands are directly assigned to the Raman-active modes characteristic of the corundum-type lattice of α-Fe_2_O_3_. The low-frequency band at 218 cm^−1^ is associated with localized lattice vibrations, whereas the peaks at 281 and 392 cm^−1^ are attributed to E_g_ and T_2g_ modes, which are linked to internal vibrations within tetrahedral and octahedral coordination sites. The sharp band at 492 cm^−1^ corresponds to Fe–O stretching vibrations in octahedral sites, while the prominent peak at 593 cm^−1^ is assigned to the A_1g_ mode. This high-frequency configuration is typical of spinel-type architectures and is frequently associated with the coexistence of localized maghemite (γ-Fe_2_O_3_) fractions alongside the α-Fe_2_O_3_ matrix [39,40,41].

The position of the main band at ~593 cm^−1^ appears slightly red-shifted and broadened compared to the standard values typically reported for macrocrystalline or bulk iron oxide references. This phenomenon is a direct consequence of the nanoscale dimensions of the crystallites, which triggers phonon confinement effects, structural surface disorder, and interfacial lattice strains. As documented by Bersani et al. [42], these structural distortions are common in nanomaterials and typically result in asymmetric broadening and shifts in Raman scatter coordinates, directly agreeing with the small physical core sizes (44.1 nm) calculated in our previous AFM section.

It is critical to highlight that zero-valent metallic silver (Ag^0^) is intrinsically inactive in first-order Raman scattering due to its symmetrical crystal selection rules, explaining why no direct, exclusive elemental silver bands appeared in the spectrum. The presence of Ag^0^ was confirmed further by XRD. However, because of its plasmonic nature, the presence of localized silver domains on the iron oxide surface plays a fundamental role via electromagnetic field enhancement. The metallic silver acts as a hotspot catalyst for surface-enhanced Raman scattering (SERS), amplifying the local electromagnetic field generated by the laser source [43]. This interfacial SERS coupling significantly boosted the scattering efficiency of the adjacent iron oxide crystal lattice, allowing the distinct and highly resolved identification of the underlying vibrational fingerprint of the hematite phase.

**Figure 5:**
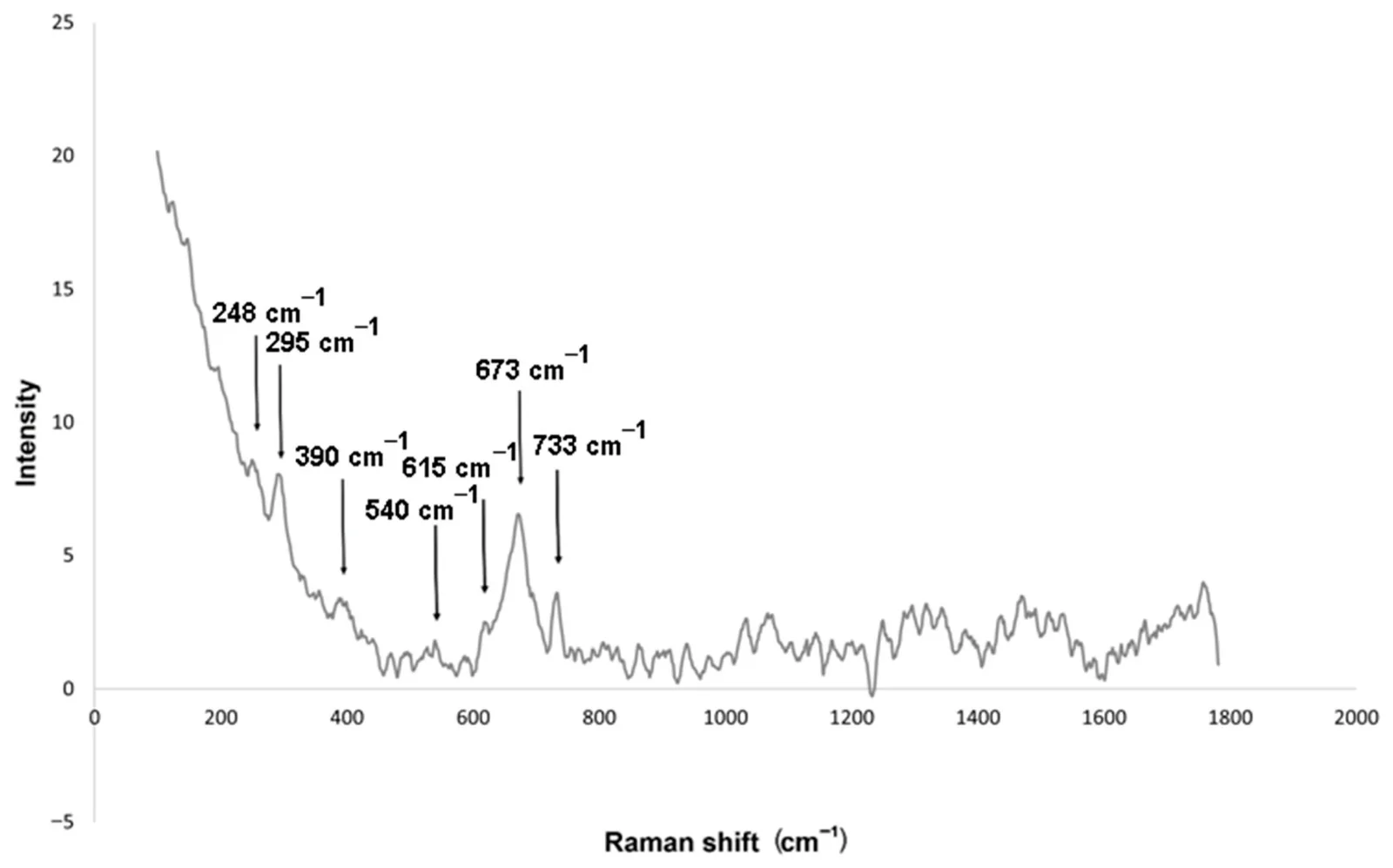
Raman scattering spectrum of CoFe bimetallic oxide nanoparticles revealing the coexistence of Co_3_O_4_ (673 cm^−1^) and cobalt ferrite CoFe_2_O_4_ spinel phases.

The structural evolution and phase composition of the bimetallic oxide nanomaterials synthesized using the sequential growth of cobalt oxide over pre-existing iron oxide seeds were comprehensively investigated using confocal Raman spectroscopy. The recorded vibrational scattering spectrum for the CoFe nanoparticles is displayed in Figure 5.

The Raman analysis of the CoFe revealed a series of highly resolved, prominent scattering bands centered at 248, 295, 390, 540, 615, 673, and 733 cm^−1^. This multiband spectral fingerprint is highly characteristic of mixed iron–cobalt transition metal oxides adopting a cubic spinel-type architecture. Mechanistically, the scattering peaks recorded at 295 and 390 cm^−1^ are assigned to the active Eg and T_2g_ vibrational modes, respectively. These modes originate from the localized out-of-phase and in-phase bending movements of Fe–O and Co–O coordination bonds distributed within both tetrahedral (T_d_) and octahedral (O_h_) sub-lattice sites. Furthermore, the intermediate band emerging at 540 cm^−1^ is directly coupled with the structural vibrations of metal-oxygen octahedral environments, while the shoulder located at 615 cm^−1^ is confidently ascribed to the characteristic A_1g_ mode of localized ferrite phases.

A key structural feature of the spectrum is the emergence of an exceptionally intense, sharp scattering band centered at ~673 cm^−1^. This high-energy vibration represents the dominant contribution of the active A_1g_ mode of crystalline cobalt oxide (Co_3_O_4_), which reflects the stretching vibrations of Co–O bonds within octahedral sites of the spinel structure. The simultaneous detection of this intense peak alongside the ferrite modes strongly highlights the coexistence of a distinct Co_3_O_4_ secondary phase intimately coupled with a cobalt ferrite (CoFe_2_O_4_) network. From a chemical synthesis perspective, this hybrid architecture can be explained by the reaction dynamics of the biogenic multi-step protocol. During the second incubation stage, the newly introduced Co^2+^ precursors interact not only with each other to yield independent Co_3_O_4_ outer domains, but also react directly with remaining, unreacted iron ion fractions trapped at the seed interfaces from the primary stage. Stimulated by the thermal energy and the reduction potential of the *A. occidentale* phytochemicals, these boundary ions undergo solid-state diffusion and chemical complexation, precipitating into the stable, magnetically coupled CoFe_2_O_4_ spinel phase.

Additionally, the appearance of the high-frequency band at 733 cm^−1^, combined with the subtle broadening and shifts in the overall spectrum compared to bulk references, underscores the nanoscale constraints of the sample. These variations are heavily driven by localized structural distortions, surface defects, oxygen vacancies, and lattice strain at the heterointerfaces. As the particle core parameters contract, aligning with the mean physical core dimension of 55.4 nm calculated using AFM, the relaxation of the phonon selection rules takes place, leading to asymmetric peak modifications.

**Figure 6:**
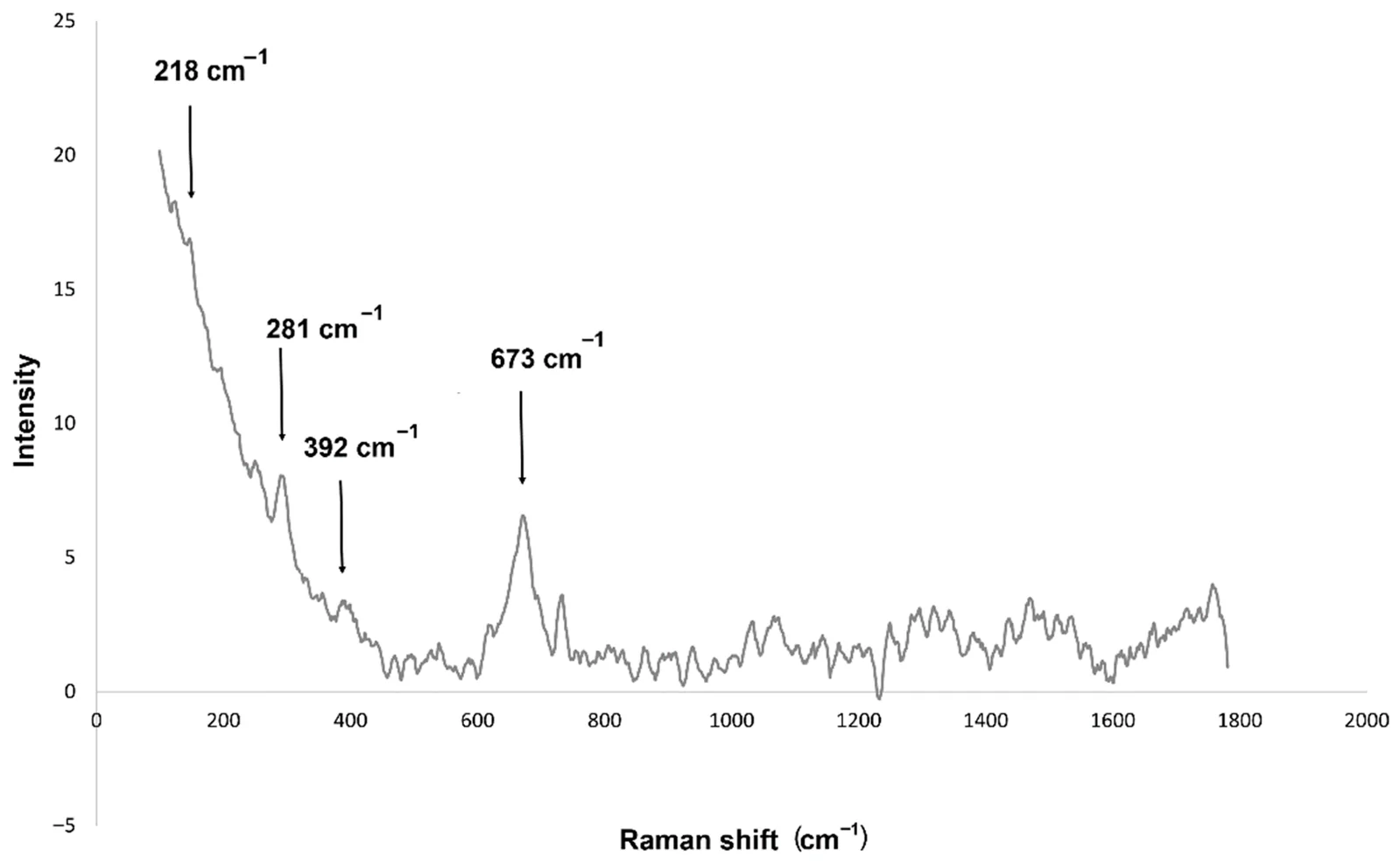
Raman scattering spectrum of CuFe bimetallic oxide nanoparticles showing hematite (α-Fe_2_O_3_) bands and the characteristic Cu_2_O mode at 144 cm^−1^.

The architectural configuration and phase preservation of the bimetallic oxide heterostructures synthesized using the deposition of copper species onto iron oxide seeds were evaluated by Raman spectroscopy. The resulting vibrational scattering spectrum for the CuFe nanoparticles is presented in Figure 6.

The Raman spectrum of the CuFe exhibited distinct scattering peaks located at 144, 216, 281, 388, and 414 cm^−1^, alongside a prominent, asymmetric broad band with an absolute maximum centered at approximately 613 cm^−1^. This specific vibrational profile reveals the coexistence of two well-defined, segregated crystalline phases, providing direct evidence of the successful assembly and preservation of the proposed bimetallic oxide heteronanostructure.

The scattering bands positioned at 216, 281, 388, and 414 cm^−1^, together with the broad feature at ~613 cm^−1^, are assigned to the active optical phonon modes of the corundum-type hematite phase (α-Fe_2_O_3_). A slight low-frequency shift (red-shift) and noticeable line broadening were observed when comparing these positions to standard macrocrystalline or bulk iron oxide references (where the primary modes typically emerge at ~225 and 290 cm^−1^). This phenomenon is widely documented in nanomaterial literature and is driven by spatial phonon confinement effects within the nanocrystals and interfacial lattice strain developed at the heterojunctions [43]. The retention of these sharp, characteristic iron oxide peaks confirms that the underlying core framework did not undergo severe phase degradation, unwanted reduction, or uncontrolled cationic interdiffusion during the second thermal deposition stage of the copper precursor.

Concurrently, the intense, well-defined peak emerging at 144 cm^−1^ serves as an unmistakable spectroscopic fingerprint of crystalline cuprous oxide (Cu_2_O). This low-frequency mode corresponds to the active Γ_15_ (F_1u_) vibrational symmetry, which is significantly enhanced in intensity due to resonance Raman scattering effects common to biogenically reduced copper oxides. It is structurally important to note that highly crystalline Cu_2_O systems also exhibit secondary multiphonon scattering overtones and combination bands located close to 218 and 415 cm^−1^. The close harmonic overlap between these secondary copper oxide overtones and the fundamental active phonon modes of the adjacent hematite lattice explains the enhanced intensity and the altered peak geometry recorded at the 216 and 414 cm^−1^ coordinates.

**Figure 7:**
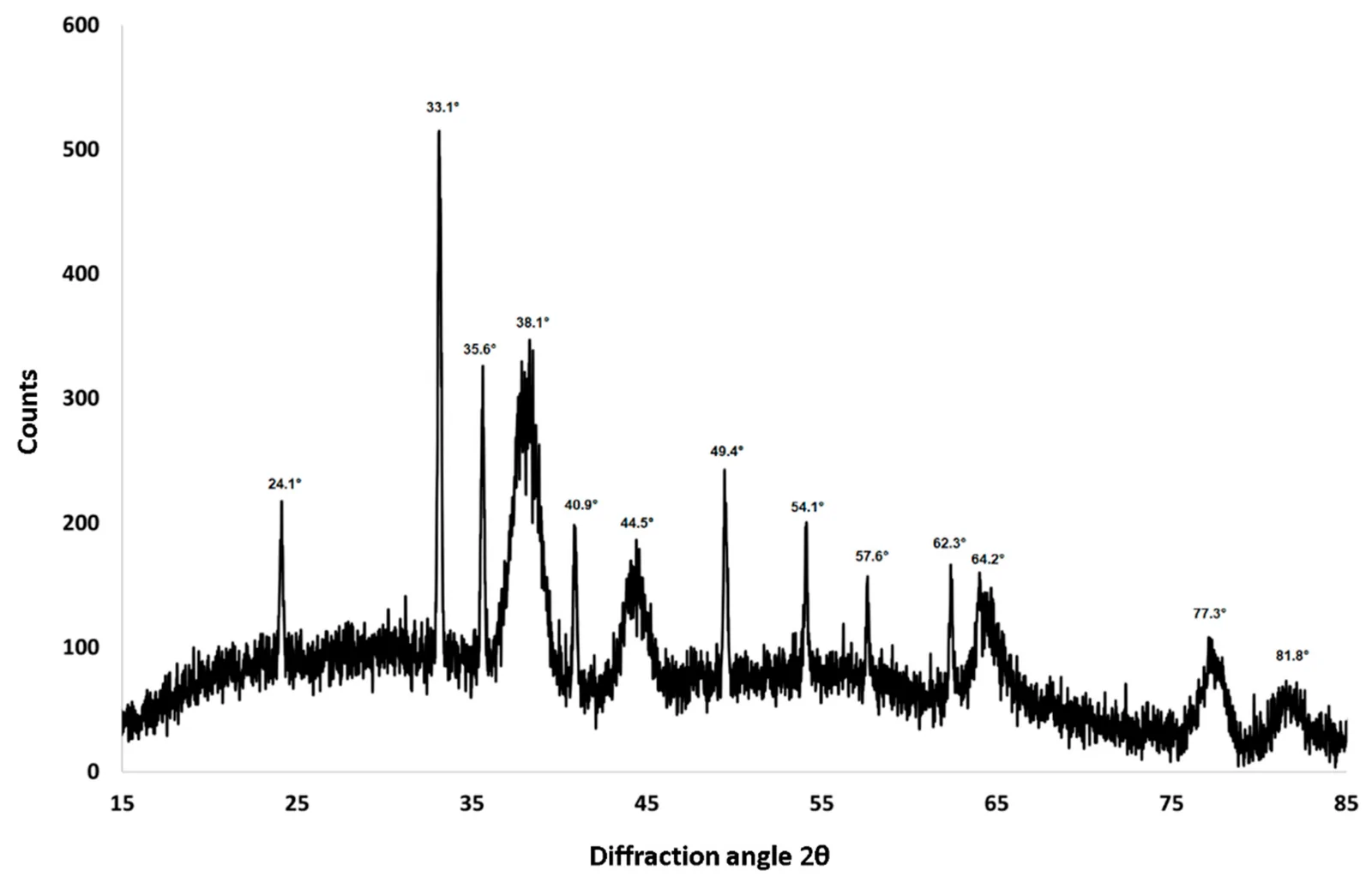
X-ray diffraction pattern of the green-synthesized AgFe bimetallic oxide nanoparticles obtained using *A. occidentale* L. pericarp extract, showing the characteristic reflections assigned to α-Fe_2_O_3_ (hematite) at 2θ = 24.1°, 33.1°, 35.6°, 40.9°, 49.4°, 54.1°, 57.6°, and 62.3°, corresponding to the (012), (104), (110), (113), (024), (116), (018)/(122), and (214) crystallographic planes, respectively. Additional reflections at 38.1°, 44.5°, 64.2°, 77.3°, and 81.8° were indexed to metallic Ag, corresponding to the (111), (200), (220), (311), and (222) planes, respectively.

**Figure 8:**
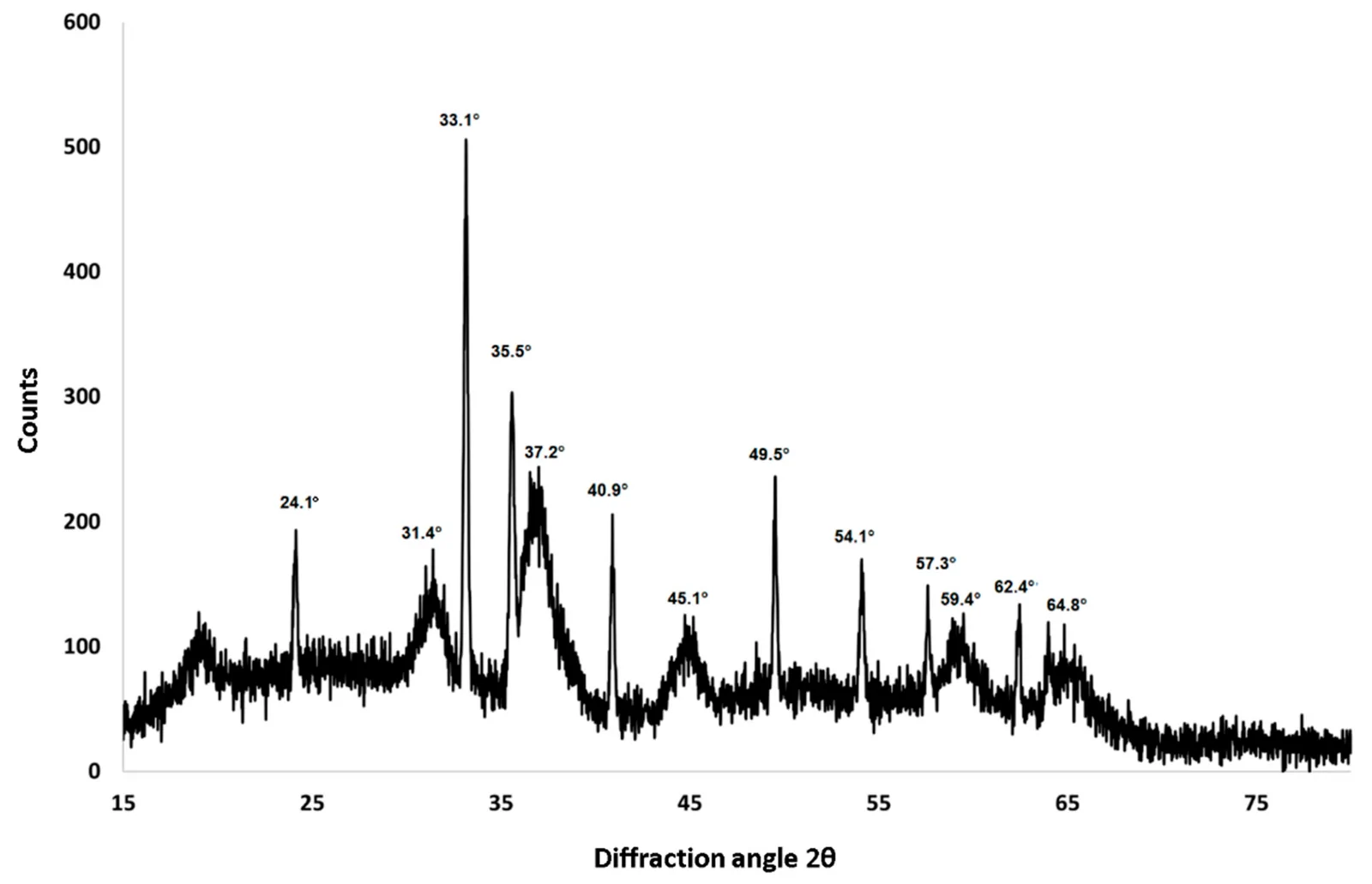
X-ray diffraction pattern of the green-synthesized CoFe bimetallic oxide nanoparticles obtained using *A. occidentale* L. pericarp extract, showing reflections attributed to α-Fe_2_O_3_ at 2θ = 24.1°, 33.1°, 40.9°, 49.5°, 54.1°, and 62.4°, together with characteristic reflections of CoFe_2_O_4_ (cobalt ferrite) at 30.2°, 35.5°, 43.3°, 57.3°, and 62.7°, indexed to the (220), (311), (400), (511), and (440) planes, respectively. Reflections at 31.4°, 37.2°, 45.1°, 59.4°, and 64.8° were assigned to Co_3_O_4_, corresponding to the (220), (311), (400), (511), and (440) planes, respectively.

**Figure 9:**
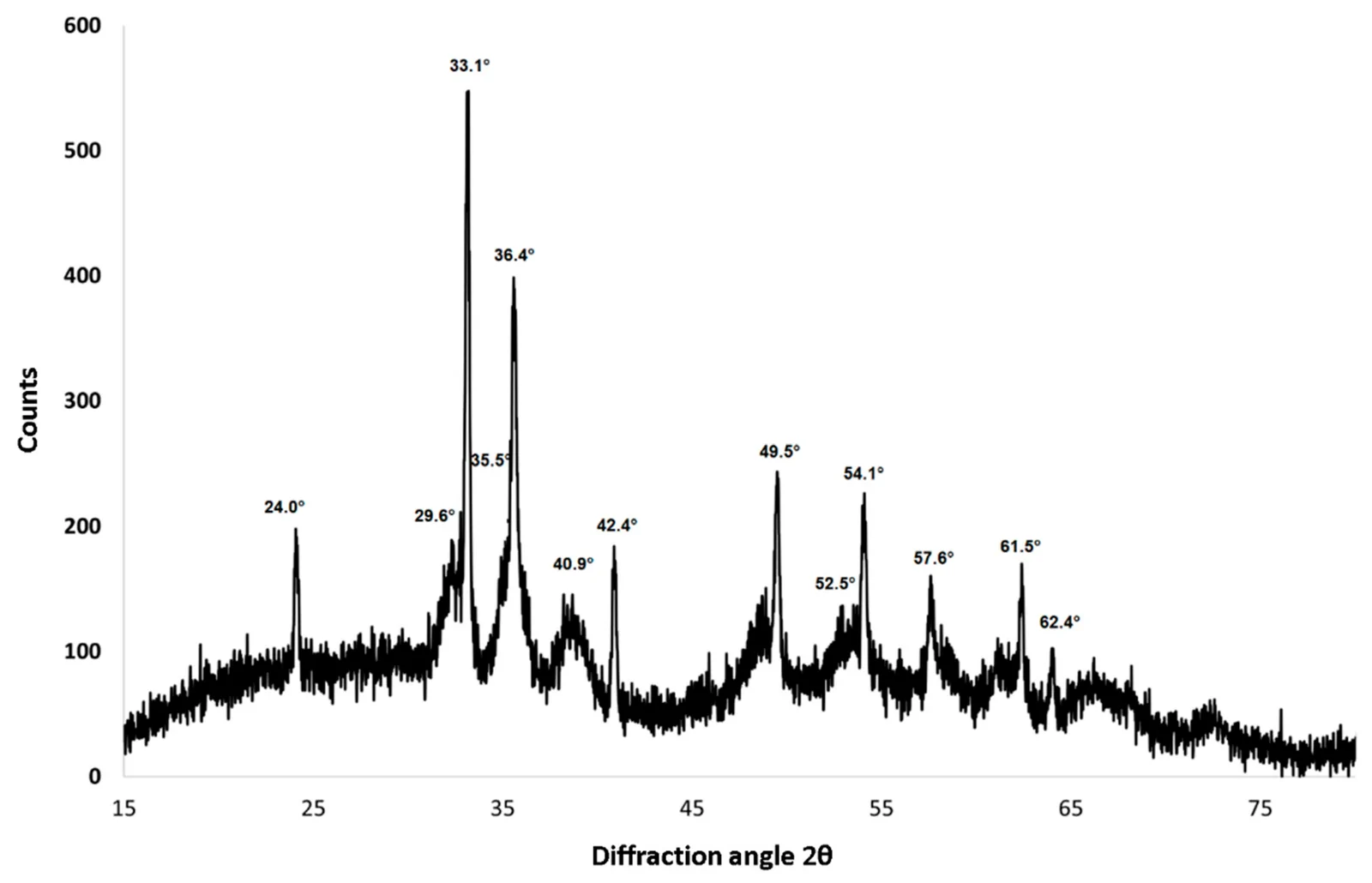
X-ray diffraction pattern of the green-synthesized CuFe bimetallic oxide nanoparticles obtained using *A. occidentale* L. pericarp extract, showing reflections assigned to α-Fe_2_O_3_ (hematite) at 2θ = 24.0°, 33.1°, 35.6°, 40.9°, 49.5°, 54.1°, 57.6°, and 62.4°, corresponding to the (012), (104), (110), (113), (024), (116), (018)/(122), and (214) crystallographic planes, respectively. Additional reflections at 29.6°, 36.4°, 42.4°, 52.5°, and 61.5° were indexed to Cu_2_O (cuprous oxide), corresponding to the (110), (111), (200), (211), and (220) planes, respectively.

The crystalline structure and phase composition of the bimetallic oxide nanoparticles were further investigated by powder X-ray diffraction (XRD). The diffractograms of AgFe, CoFe, and CuFe are shown in Figure 7, Figure 8 and Figure 9. All patterns exhibit broadened Bragg peaks, characteristic of nanocrystalline materials, with no evidence of large-scale amorphous domains.

For the AgFe system, the diffraction peaks at 2θ = 38.1°, 44.5°, 64.2°, 77.3° and 81.8° are unambiguously indexed to the (111), (200), (220), (311) and (222) planes of face-centred cubic metallic silver. This confirms the presence of zero-valent Ag^0^ in the AgFe system. The remaining reflections at 24.1°, 33.1°, 35.6°, 40.9°, 49.4°, 54.1°, 57.6° and 62.3° match the rhombohedral hematite phase (α-Fe_2_O_3_), confirming the biphasic nature of the AgFe bimetallic oxide nanostructures, in full agreement with the Raman bands assigned to hematite (Figure 4). No additional peaks from silver oxides were detected, indicating that the phytochemicals effectively reduced Ag^+^ to metallic Ag^0^ while preserving the iron oxide core. The presence of metallic silver (Ag^0^) alongside hematite (α-Fe_2_O_3_) is a key structural feature. Similar biphasic Ag/α-Fe_2_O_3_ heterostructures have been described by Singh et al. [12] and Yaqoob et al. [11], who demonstrated that the plasmonic silver domains enhance the catalytic performance via interfacial electron transfer. The absence of silver oxide (Ag_2_O) peaks indicates that the polyphenols from the *A. occidentale* extract were sufficiently reductive to achieve complete conversion of Ag^+^ to Ag^0^, a behavior that aligns with the well-documented reducing capacity of cashew phytochemicals [14].

For the CoFe sample, the pattern shows a more complex set of reflections. The peaks at 2θ ≈ 30.2° (shoulder at 31.4°), 35.5°, 37.2°, 43.3° (band centred at 45.1°), 57.3° (band at 59.4°) and 62.7° (band at 64.8°) are characteristic of a cubic spinel structure. The prominent reflection at 35.5° and the doublet around 57–59° are consistent with the (311) and (511)/(440) planes of cobalt ferrite (CoFe_2_O_4_), while the shoulders at 31.4°, 37.2° and 45.1° can be assigned to the (220), (311) and (400) planes of Co_3_O_4_. The additional low-intensity peaks at 24.1°, 33.1°, 40.9°, 49.5°, 54.1° and 62.4° likely arise from residual hematite or from the iron-rich domains of the spinel solid solution. This multiphase composition corroborates the Raman data (Figure 5), which revealed the coexistence of Co_3_O_4_ and cobalt ferrite phases.

In the CuFe diffractogram, the peaks at 2θ = 29.6°, 36.4° (observed as a shoulder near 35.6°), 42.4° (overlapped with the hematite 40.9° peak), 52.5° (merged with the 54.1° doublet) and 61.5° (close to the 62.4° peak) are attributed to cubic cuprous oxide (Cu_2_O). The remaining reflections at 24.0°, 33.1°, 35.6°, 40.9°, 49.5°, 54.1°, 57.6° and 62.4° are again consistent with the hematite phase. The close overlap of several Cu_2_O and hematite peaks (e.g., the pair at 33–35° and the doublets around 49–54°) explains the broad, asymmetric bands observed in the experimental pattern. These findings are in excellent agreement with the Raman spectrum (Figure 6), which showed the characteristic Cu_2_O mode at 144 cm^−1^ alongside the hematite bands.

The mean crystallite sizes, calculated using the Scherrer equation applied to the most intense reflection of each dominant phase, were 40.7 nm (AgFe, using the Ag (111) peak), 38.8 nm (CoFe, using the CoFe_2_O_4_ (311) peak) and 35.6 nm (CuFe, using the Cu_2_O (111) peak). These values are consistently smaller than the physical particle diameters determined by AFM (44.1, 55.4 and 41.5 nm for AgFe, CoFe and CuFe, respectively). This discrepancy is widely reported for biogenically synthesized nanoparticles, where the phytochemical capping layer promotes the aggregation of primary crystallites into larger secondary particles during solvent evaporation for AFM imaging [12,13]. Furthermore, the crystallite dimensions obtained here are in excellent agreement with those reported for iron-based nanoparticles synthesized using other plant extracts, which typically range from 20 to 50 nm [14,39].

Overall, the XRD results confirm the successful formation of the targeted bimetallic oxide architectures, with the secondary metals (Ag, Co, Cu) adopting either metallic (Ag^0^) or oxide (Co_3_O_4_, CoFe_2_O_4_, Cu_2_O) phases that coexist with the iron oxide matrix. The crystallite sizes and phase purity are fully consistent with the Raman and AFM data, reinforcing the conclusion that the *A. occidentale* extract acts as an effective reducing and stabilising agent, yielding nanocrystalline materials with tailored structural properties.

The presented green synthesis approach and physicochemical characterization established that the *A. occidentale* phytochemicals act not merely as passive capping agents, but as active redox interfaces, leading to the formation of bimetallic oxide nanostructures. The preservation of the organic polyphenolic shell (evidenced by FTIR and the negative Zeta potential) and the intrinsic redox versatility of the mixed-oxide phases (hematite, cobalt ferrite, and cuprous oxide identified by Raman spectroscopy) are structural prerequisites that govern electron-transfer phenomena. To translate these structural features into functional biological relevance, the intrinsic radical-scavenging capacity of these bimetallic architectures was consequently evaluated using antioxidant assays.

### 3.3. Total Phenolic Content and Antioxidant Profile of the Nanoparticles

To evaluate the chemical involvement of *A. occidentale* secondary metabolites in the structural capping of the bimetallic oxide systems, as well as to determine the biological and radical-trapping performance of the distinct materials, the DPPH radical scavenging activity and FRAP ferric reducing antioxidant power were systematically monitored.

**Figure 10:**
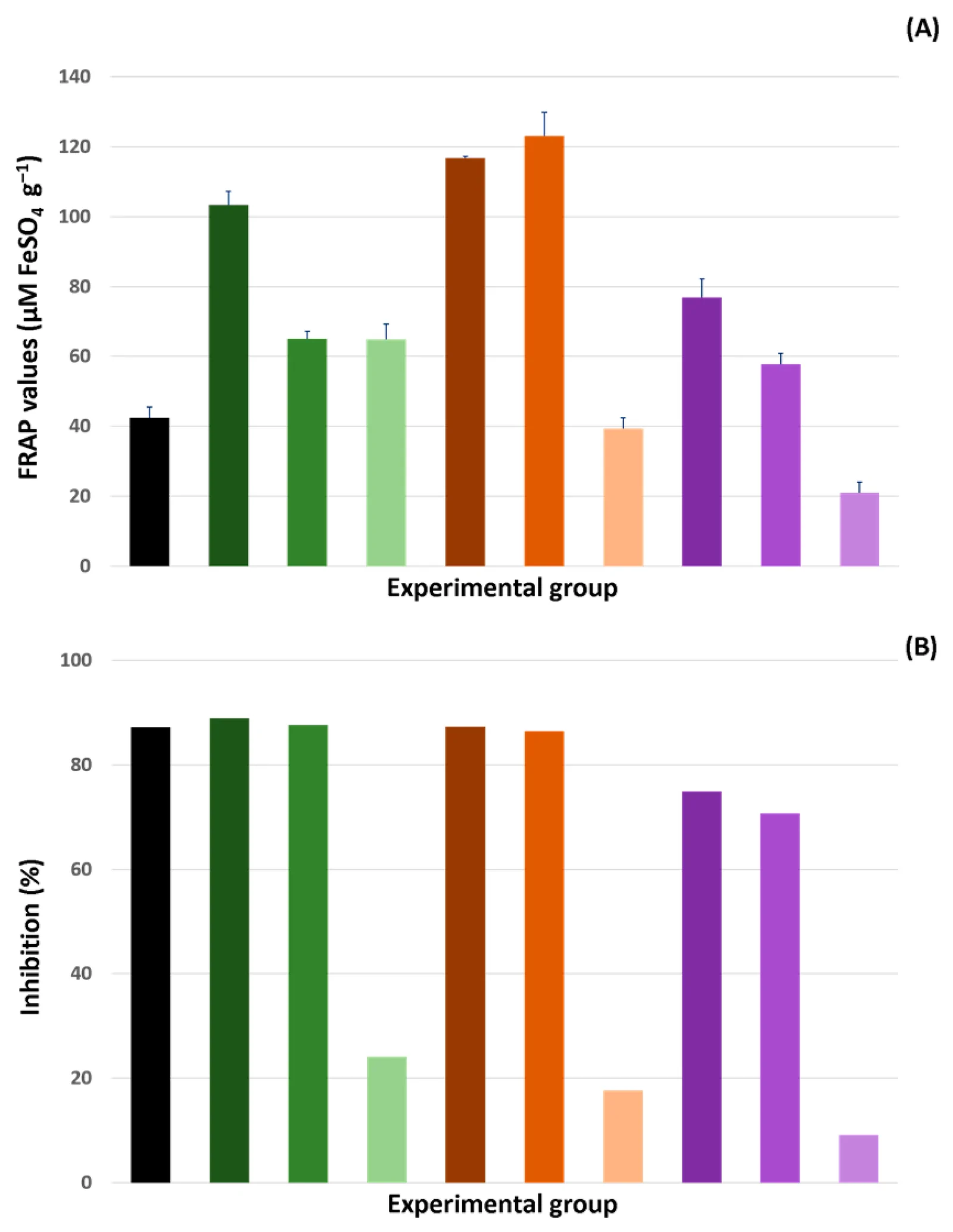
FRAP assay (**A**) and DPPH antioxidant profile assays (**B**) of the *A. occidentale* extract (black) and the synthesized AgFe, CoFe, and CuFe bimetallic oxide nanoparticle fractions. Color code: black bars, crude aqueous extract; dark green bars, as-synthesized AgFe; green bars, AgFe supernatant; light green bars, washed AgFe; brown bars, as-synthesized CoFe; orange bars, CoFe supernatant; peach bars, washed CoFe; purple bars, as-synthesized CuFe; violet bars, CuFe supernatant; light purple bars, washed CuFe.

The DPPH and FRAP assays quantify the cumulative reducing capacity of the entire colloidal suspension, encompassing contributions from the metallic oxide core, the surface-bound organic corona, and residual soluble phytochemicals. Interpreting these biological profiles as intrinsic nanomaterial properties therefore requires prior physicochemical deconvolution. The quantitative profiles obtained for the crude extract, the unwashed (as-synthesized) bimetallic oxide nanoparticles, the washed nanoparticles, and their respective reaction supernatants (SN) are compiled in Figure 10A,B.

The FRAP assay relies on electron transfer mechanisms (reducing a ferric-TPTZ complex and phosphomolybdic-phosphotungstic acids, respectively). By introducing exogenous, highly concentrated transition metal salt controls containing iron precursors (FeSO_4_), the reaction medium is flooded with active, non-complexed Fe^2+^ ions. This iron excess directly transfers electrons to the colorimetric probes, driving the chemical equilibrium forward and causing an artificial increase in absorbance that mimics a large reducing capacity.

The baseline antioxidant profile of the raw *A. occidentale* pericarp extract demonstrated a robust biological performance, with a reducing power (FRAP) of 42.38 µM FeSO_4_ g^−1^, a high density of native polyphenols (377.59 mg GAE g^−1^), and a high DPPH radical scavenging inhibition of 87.13%.

When the as-synthesized bimetallic oxide nanoparticles were evaluated, a significant synergistic enhancement associated to the extract in reducing power was observed across all three systems. The as-synthesized AgFe, CoFe, and CuFe formulations showed elevated FRAP values (103.34, 116.78, and 76.91 µM FeSO_4_ g^−1^) that comfortably surpassed the crude extract. Concurrently, their DPPH scavenging efficiencies remained highly competitive (88.89%, 87.28%, and 74.85%, respectively). This synergistic performance originates from the copresence of the reactive transition-metal core surfaces alongside an excess of unbound or weakly attached plant polyphenols that act as a dynamic stabilization network.

The true biogenic success of the synthesis is confirmed by the behavior of the samples after the washing protocols. When the nanoparticles were subjected to sequential centrifugation steps with ultrapure water, the loosely bound organic molecules and unreacted metal ions were removed from the suspension. As expected, the washed nanoparticle systems showed a controlled decrease across all analytical parameters. The washed AgFe retained a stable FRAP reducing power of 64.92 µM FeSO_4_ g^−1^ and a DPPH inhibition of 24.12%. The CoFe and CuFe followed similar trends, dropping to DPPH inhibition levels of 17.69% and 9.21%, respectively.

This drop in the inhibition percentage is mechanistically sound. The removal of the free phytochemical matrix isolates the intrinsic antioxidant profile of the permanently attached organic capping shell that forms the protective boundary of the nanostructures. The deprotonated phenolic groups strictly bound to the bimetallic oxide surfaces cannot be removed by washing. These immobilized groups remain available at the solid-liquid interface to deactivate reactive species, allowing the clean nanomaterials to preserve a steady, localized antioxidant activity.

The reaction supernatants (SN) represent the chemical components left behind in the liquid phase after the nanoparticles precipitate. The high levels of FRAP and DPPH inhibition found in the supernatants, such as the 87.57% and 86.40% radical scavenging rates for the AgFe and CoFe systems, mirror the baseline behavior of the crude plant extract.

This persistent high activity in the residual liquid suggests that the initial *A. occidentale* extract was added in a well-calculated thermodynamic excess. This stoichiometric excess ensured that a sufficient amount of active biomolecules was available to entirely reduce the metal cores and provide steric stabilization, while the remaining antioxidant compounds were left unaltered in the liquid phase to prevent uncontrolled particle agglomeration. Ultimately, these combined evaluations confirm that the green synthesis protocol successfully anchors the natural antioxidant properties of cashew metabolites onto structurally stable bimetallic oxide platforms.

To contextualize the antioxidant activity of the synthesized bimetallic oxide nanoparticles, the DPPH radical-scavenging and ferric reducing antioxidant power (FRAP) results obtained in this study were compared with data reported for other green-synthesized bimetallic oxide nanoparticle systems (Table 2).

The comparison with previously reported bimetallic oxide nanoparticles indicates that the antioxidant activity observed in the present study falls within the range reported for other plant-mediated systems. For example, the Mn–Cu nanoparticles synthesized using Vinca rosea exhibited 51.44% DPPH inhibition at 1000 µg/mL, whereas ZnO–CuO nanoparticles synthesized using *Artemisia abyssinica* reached 95.71% inhibition at 200 µg/mL. The AgFe value obtained in the present study (88.88%) was therefore higher than that reported for Mn–Cu and approached the value reported for ZnO–CuO, although direct quantitative comparisons should be interpreted with caution because the assays were performed under different experimental conditions and nanoparticle concentrations. In addition, AgPt nanoalloys synthesized using *Vernonia mespilifolia* showed a DPPH IC_50_ of 19.5 µg/mL, further illustrating the diversity of antioxidant responses reported for green-synthesized bimetallic nanomaterials.

For the FRAP assay, the AgFe, CoFe, and CuFe bimetallic oxide nanoparticles presented reducing capacities of 103.34, 116.78, and 76.91 µM FeSO_4_ g^−1^, respectively. Although the FRAP results reported in the literature are expressed using different units and experimental conditions, the data provide a useful comparative context for the reducing activity of the nanoparticles investigated herein. Overall, the antioxidant activity observed for the AgFe and CuFe systems suggests that their performance may result from the combined contribution of the iron oxide phase, the secondary metal, and the phytochemical layer associated with the nanoparticle surface. Importantly, the antioxidant activity measured after washing the nanoparticles is consistent with the contribution of a strongly associated phytochemical corona that remains at the nanoparticle surface rather than reflecting solely the activity of freely dissolved extract constituents.

### 3.4. Catalytic Performance Evaluation (Reduction of 4-Nitrophenol)

The antioxidant assays confirmed that the biogenic bimetallic oxide nanostructures possess a remarkable capacity to mediate rapid electron transfer to exogenous radical probes. This electron-donor capacity is intrinsically linked to the ability to act as heterogeneous redox catalysts in environmental contexts. Since both antioxidant scavenging and pollutant degradation rely on the availability of surface-active sites and the electronic coupling between the bimetallic phases, the benchmark catalytic reduction of 4-nitrophenol (4-NP) was selected to quantify the efficiency of interfacial electron transfer.

Unlike molecular antioxidants (e.g., BHT or Trolox), which are consumed stoichiometrically during radical neutralization, the bimetallic oxide nanoparticles exhibit a nonenzyme-like behavior. The inorganic cores catalyze the continuous regeneration of electron-donating species at the surface via reversible redox cycling, ensuring a sustained scavenging effect without rapid depletion of the capping ligands, a feature highly desirable for long-term biomedical and environmental applications.

The environmental viability and practical functionality of the synthesized bimetallic oxide nanomaterials were assessed through the catalytic reduction of 4-NP to 4-aminophenol (4-AP), a benchmark reaction for industrial wastewater remediation. The reaction was conducted in an aqueous medium using NaBH_4_ as a reducing agent. Given that NaBH_4_ was maintained in significant thermodynamic excess relative to 4-NP, the reaction kinetics were modeled according to a pseudo-first-order regime, allowing for the determination of the apparent rate constant (k_obs_). The kinetic and catalytic efficiency parameters for the three bimetallic oxide systems and the Fe_2_O_3_ control are summarized in Table 3.

Comparative analysis reveals that the secondary metal composition exerts precise control over the thermodynamic efficiency and catalytic reaction velocity. To establish a baseline performance, Fe_2_O_3_ nanoparticles obtained in the first step of the synthesis were evaluated as a monometallic control, exhibiting sluggish reaction kinetics (k_obs_ = 1.6 × 10^−3^ s^−1^; reduction time = 41 min). The copper-based bimetallic oxide system (CuFe) demonstrated exceptional catalytic performance, achieving near-total conversion (99.1 ± 0.5%) within the shortest timeframe (6.3 ± 0.6 min). This superior performance is quantitatively supported by its apparent rate constant (k_obs_ = 17.0 × 10^−3^ s^−1^), which is nearly double the speed observed for the other systems. The elevated catalytic activity of CuFe can be attributed to synergistic electronic effects at the heterointerface identified using Raman spectroscopy, where the cuprous oxide phase (Cu_2_O) acts as an efficient electron relay. Copper possesses an ideal adsorption energy for nitro groups, facilitating rapid electron transfer from the ${BH}_{4}^{-}$ donors to the 4-NP acceptors.

The CoFe system displayed intermediate performance, concluding the reaction in 11.3 ± 0.5 min with an efficiency of 93.9 ± 1.5% and a k_obs_ of 9.4 × 10^−3^ s^−1^. While the complex spinel and cobalt ferrite (CoFe_2_O_4_) network provides excellent structural stability, it exhibits slightly slower electron transfer kinetics in borohydride-mediated reactions compared to the copper-based interface.

Finally, the AgFe NPs displayed the longest reduction time (26.7 ± 1.1 min) and the lowest k_obs_ (9.1 × 10^−3^ s^−1^) among NPs, despite achieving a high final conversion (96.1 ± 2.4%). Although silver is classically recognized for its potent catalytic power, the AFM and DLS results revealed that this system presents a compact physical cores wrapped in a moderate hydration layer (as indicated by the negative Zeta potential of −17.5 mV). This robust phytochemical barrier, composed of the *A. occidentale* polyphenols, is excellent for long-term colloidal stability and antioxidant activity, but it generates steric hindrance that limits reagent access to the active metal surface, thereby prolonging reaction times.

Overall, the 4-nitrophenol reduction assay validates the multifunctionality of the developed bimetallic oxide nanomaterials. The organic coating derived from the *A. occidentale* pericarp not only confers excellent antioxidant properties for radical scavenging and prevents aggregation during catalytic cycles but also stabilizes bimetallic oxide architectures that serve as potent environmental remediation agents, fully aligned with green chemistry and circular economy principles.

To contextualize the results, the comparison of the catalytic performance of our bimetallic oxide nanoparticles with other green-synthesized bimetallic systems reported in the literature is shown in Table 4.

As summarized in Table 4, the catalytic performance of the bimetallic nanoparticles synthesized in this work followed the order CuFe > CoFe > AgFe under identical experimental conditions (0.5 mM 4-NP and 50 mM NaBH_4_). The CuFe system exhibited superior catalytic activity, achieving a rate constant (k_obs_ of 17.0 × 10^−3^ s^−1^) and completing 4-NP reduction in just 6.3 min. This result outperforms biogenic systems reported in the literature, such as Ag–Fe nanoparticles synthesized using *Salvia officinalis* (1.1 × 10^−3^ s^−1^; 45 min [47] and Cu–Ag/Cu–Ni nanoparticles from ginger rhizome (4.05 − 6.08 ×10^−3^ s^−1^; 10 min) [50].

Furthermore, the CuFe formulation surpassed Au–Ag nanoparticles obtained from *Punica granatum* extract (13.3 × 10^−3^ s^−1^) [49], despite employing lower concentrations of NaBH_4_ (50 mM vs. 100 mM). The enhanced activity across the series, particularly for CuFe, can be attributed to favorable synergistic electronic interactions between the constituent metals, which accelerate electron transfer from the ${BH}_{4}^{-}$ donor to the 4-NP acceptor [48,50]. These findings confirm that cashew pericarp waste serves as an effective precursor for synthesizing high-performance, low-cost bimetallic catalysts that rival or exceed noble-metal counterparts.

## 4. Conclusions

This investigation shows that the secondary transition metal component is the critical determinant for tuning the dual biological and catalytic functionality of green-synthesized iron-based bimetallic oxide nanostructures. Comparative analysis confirms that AgFe, CoFe, and CuFe formulations exhibit distinct redox profiles, enabling tailored responses to both reactive oxygen species in biological environments and refractory organic pollutants in industrial wastewater. Utilizing *A. occidentale* extracts provides a sustainable, low-cost synthetic route that ensures high stability and biocompatibility without the use of hazardous reagents. The enhanced catalytic toward 4-nitrophenol reduction and antioxidant performance of the bimetallic oxide systems, compared to the iron oxide control, is consistent with a synergistic contribution from the secondary metal oxide phases, although the exact mechanism requires further investigation. These results establish a versatile framework for the design of multifunctional bimetallic oxide nanoparticles, offering a robust strategy for integrating environmental remediation with biomedical protection. Future applications of these materials hold significant promise for advancing high-performance, eco-friendly systems in both clinical diagnostics and industrial water treatment.

## Figures and Tables

**Table 1:** Hydrodynamic diameter dH, polydispersity index (PdI), and Zeta potential (Zp) of the bimetallic oxide nanoparticles synthesized using *A. occidentale* L. pericarp extract.

Nanoparticle System	d_H_ (Z-Average, nm)	PdI	Zp (ζ, mV)
AgFe	193.7 ± 23.2	0.375 ± 0.02	−17.5 ± 0.8
CoFe	317.7 ± 13.0	0.573 ± 0.01	−16.3 ± 1.5
CuFe	205.2 ± 20.2	0.470 ± 0.05	−8.6 ± 2.6

**Table 2:** Comparison of antioxidant activity reported for green-synthesized bimetallic oxide nanoparticles.

Nanoparticle	Extract	DPPH Inhibition	FRAP	Ref.
AgFe (as-synthesized)	cashew extract	88.89%	103.34 µM FeSO_4_ g^−1^	This work
CoFe (as-synthesized)	cashew extract	87.28%	116.78 µM FeSO_4_ g^−1^	This work
CuFe (as-synthesized)	cashew extract	74.85%	76.91 µM FeSO_4_ g^−1^	This work
AgPt nanoalloy	*Vernonia mespilifolia*	IC_50_ 19.5 µg/mL	44.1 mg GAE g^−1^	[44]
Mn–Cu	*Vinca rosea*	51.44% (1000 µg/mL)	Abs 0.176 (1 mg/mL)	[45]
ZnO–CuO	*Artemisia abyssinica*	95.71% (200 µg/mL)	Abs 1.826 (200 µg/mL)	[46]

**Table 3:** Catalytic performance parameters for the reduction of 4-NP mediated by the A. occidentale-stabilized bimetallic oxide nanoparticles and the Fe_2_O_3_ control.

Catalyst Sample	Reduction Time (min)	Rate Constant, k_obs_ (×10^−3^ s^−1^)	Conversion Efficiency (%)
AgFe	26.7 ± 1.1	9.1 ± 0.5	96.1 ± 2.4
CoFe	11.3 ± 0.5	9.4 ± 0.2	93.9 ± 1.5
CuFe	6.3 ± 0.6	17.0 ± 1.7	99.1 ± 0.5
Fe_2_O_3_ (Control)	41.0 ± 1.8	1.6 ± 0.9	93.1 ± 1.6

**Table 4:** Comparison of the catalytic performance of bimetallic oxide nanoparticles reported in the literature and in the present study for the reduction of 4-nitrophenol (4-NP) using NaBH_4_, including feedstock, metallic composition, 4-NP and NaBH_4_ concentrations, reduction time, observed rate constant (kobs), and corresponding reference.

Feedstock	Metal	4NP Conc. (mM)	NaBH_4_ Conc. (mM)	Time (min)	k_obs_ (×10^−3^ s^−1^)	Ref.
*Salvia officinalis*	Ag-Fe	0.2	20	45	1.1 *	[47]
Waste Tea Leaves	Ag-Au	0.1	NR	6-7	NR	[48]
*Punica granatum*	Au-Ag	5.0	100	6	13.3 *	[49]
Ginger rhizome	Cu-Ag/Cu-Ni	1.0	500	10	4.05–6.08	[50]
This work	AgFe	0.5	50	26.7	9.1	-
This work	CoFe	0.5	50	11.3	9.4	-
This work	CuFe	0.5	50	6.3	17.0	-
This work	Fe_2_O_3_	0.5	50	41.0	1.6	-

* Values recalculated for uniformity in the corresponding units with respect to other reports. NR = Not reported.

## Data Availability

The original contributions presented in the study are included in the article. Further inquiries can be directed to the corresponding author.

## Supplementary Materials

The following supporting information can be downloaded at: https://www.mdpi.com/article/10.3390/antiox15101363/s1, Figure S1: DLS size distribution histograms of the green-synthesized bimetallic oxide nanoparticles: (a) AgFe, (b) CoFe, and (c) CuFe. Measurements were performed in triplicate at 25 °C using a He–Ne laser (λ = 632.8 nm) and a fixed scattering angle of 173°. The multimodal profiles indicate the presence of distinct particle populations in aqueous suspension.

## Abbreviations

The following abbreviations are used in this manuscript:

4-AP	4-aminophenol
4-NP	4-nitrophenol
AgFe	Silver–Iron bimetallic oxide nanoparticles
AFM	Atomic Force Microscopy
ATR	Attenuated Total Reflectance
BHT	Butylated Hydroxytoluene
BNPs	Bimetallic Oxide Nanoparticles
CCD	Charge-Coupled Device
CM	Salt Control
CNPAT	Embrapa Tropical Agroindustry
CoFe	Cobalt–Iron bimetallic oxide nanoparticles
CuFe	Copper–Iron bimetallic oxide nanoparticles
DLS	Dynamic Light Scattering
DPPH	2,2-diphenyl-1-picrylhydrazyl
FeONPs	Iron-Oxide Nanoparticles
FTIR	Fourier-Transform Infrared Spectroscopy
FRAP	Ferric Reducing Antioxidant Power
GAE	Gallic Acid Equivalent
LNANO	Laboratory of Nanobiotechnology
NaBH_4_	Sodium Borohydride
PdI	Polydispersity Index
QE	Quercetin Equivalent
ROS	Reactive Oxygen Species
SN	Supernatant
SPR	Surface Plasmon Resonance
TBHQ	Tert-Butylhydroquinone
TFC	Total Flavonoid Content
TPC	Total Phenolic Content
UV-Vis	Ultraviolet–Visible
XRD	X-ray Diffraction
Zp	Zeta Potential
