# Supplementary material for: From Agro-Industrial Waste to Multifunctional Nanomaterials: Antioxidant and Catalytic Properties of Iron-Based Bimetallic Nanoparticles Synthesized with Anacardium occidentale Pericarp Extract
Source: Antioxidants (Basel). 2026 Oct 21;15(10):1363. doi: 10.3390/antiox15101363 (PMC13701183; doi:10.3390/antiox15101363)

Supplementary Materials

Figure S1. DLS size distribution histograms of the green-synthesized bimetallic oxide nanoparticles: (a) AgFe, (b) CoFe, and (c) CuFe. Measurements were performed in triplicate at 25 °C using a He–Ne laser (λ = 632.8 nm) and a fixed scattering angle of 173°. The multimodal profiles indicate the presence of distinct particle populations in aqueous suspension.

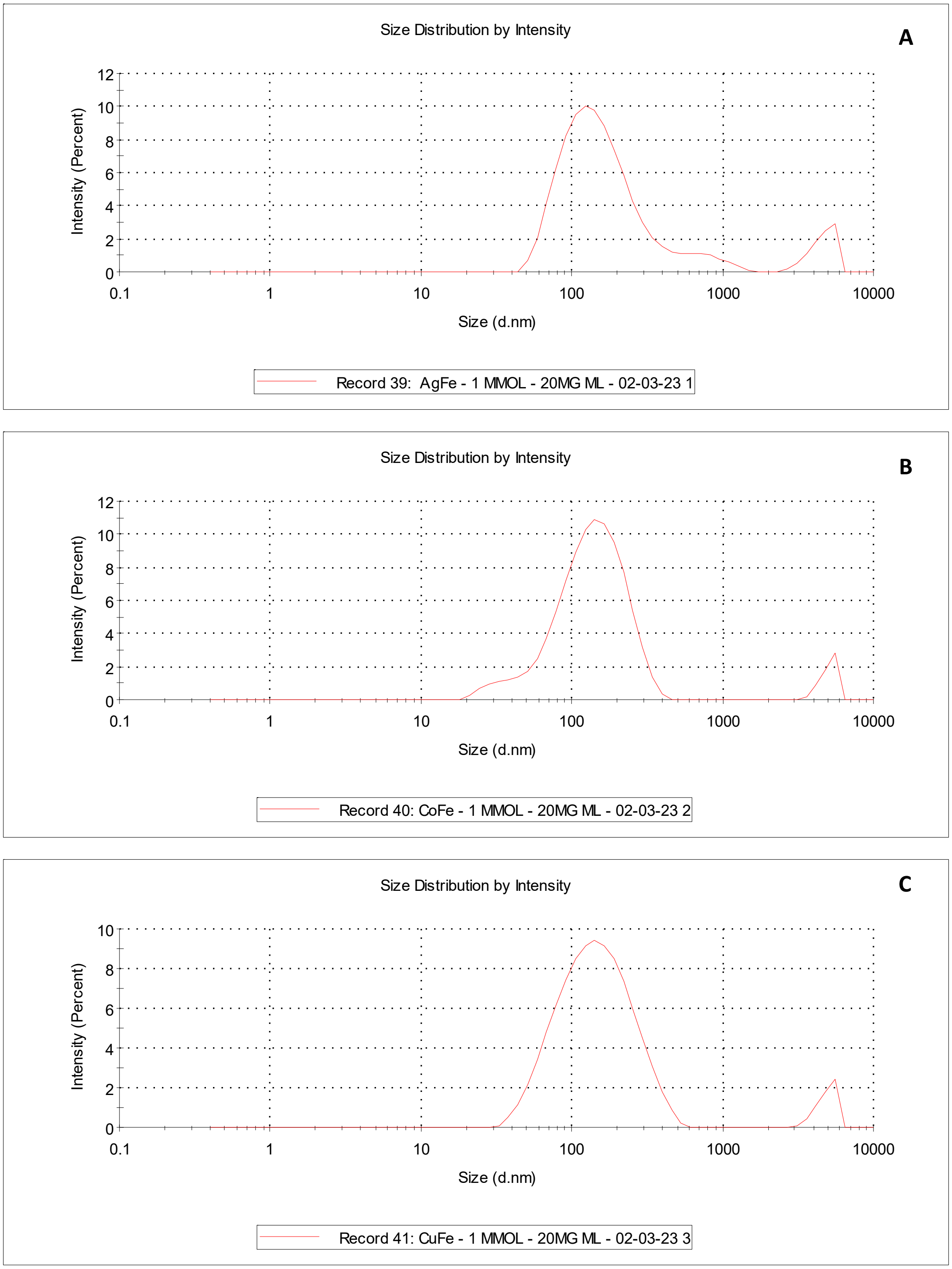

Supplement: Supplementary file 1 [file antioxidants-15-01363-s001.zip › antioxidants-4507151-supplementary.pdf]
